# Safety and Efficacy of Combined Intramuscular/Intranasal RAZI-COV PARS Vaccine Candidate Against SARS-CoV-2: A Preclinical Study in Several Animal Models

**DOI:** 10.3389/fimmu.2022.836745

**Published:** 2022-05-26

**Authors:** Seyed Reza Banihashemi, Ali Es-haghi, Mohammad Hossein Fallah Mehrabadi, Mojtaba Nofeli, Ali Rezaei Mokarram, Alireza Ranjbar, Mo Salman, Monireh Hajimoradi, Seyad Hossein Razaz, Maryam Taghdiri, Mohsen Bagheri, Maryam Dadar, Zuhair Mohammad Hassan, Mohammad Eslampanah, Zahra Salehi Najafabadi, Mohsen Lotfi, Akbar Khorasani, Fereidoon Rahmani

**Affiliations:** ^1^ Department of immunology, Razi Vaccine and Serum Research Institute, Agricultural Research, Education and Extension Organization (AREEO), Karaj, Iran; ^2^ Department of Physico Chemistry, Razi Vaccine and Serum Research Institute, Agricultural Research, Education and Extension Organization (AREEO), Karaj, Iran; ^3^ Department of Epidemiology, Razi Vaccine and Serum Research Institute, Agricultural Research, Education and Extension Organization (AREEO), Karaj, Iran; ^4^ Department of Research and Development, Razi Vaccine and Serum Research Institute, Agricultural Research, Education and Extension Organization (AREEO), Karaj, Iran; ^5^ Department of Quality Assurance, Razi Vaccine and Serum Research Institute, Agricultural Research, Education and Extension Organization (AREEO), Karaj, Iran; ^6^ Clinic of Pediatrics, Institute of Interventional Allergology and Immunology, Bonn, Germany; ^7^ Animal Population Health Institute of College of Veterinary Medicine and Biomedical Sciences, Colorado State University, Fort Collins, CO, United States; ^8^ Department of Immunology, School of Medical Sciences, Tarbiat Modares University, Tehran, Iran; ^9^ Department of Pathology, Razi Vaccine and Serum Research Institute, Agricultural Research, Education and Extension Organization (AREEO), Karaj, Iran; ^10^ Department of Quality Control, Razi Vaccine and Serum Research Institute, Agricultural Research, Education and Extension Organization (AREEO), Karaj, Iran

**Keywords:** RAZI-COV PARS, COVID-19, SARS-CoV-2 preclinical study, vaccine, spike protein

## Abstract

Several vaccine candidates for COVID-19 have been developed, and few vaccines received emergency approval with an acceptable level of efficacy and safety. We herein report the development of the first recombinant protein-based vaccine in Iran based on the recombinant SARS-CoV-2 spike protein in its monomeric (encompassing amino acid 1-674 for S1 and 685-1211 for S2 subunits) and trimer form (S-Trimer) formulated in the oil-in-water adjuvant system RAS-01 (Razi Adjuvant System-01). The safety and immunity of the candidate vaccine, referred to as RAZI-COV PARS, were evaluated in Syrian hamster, BALB/c mice, Pirbright guinea pig, and New Zeeland white (NZW) rabbit. All vaccinated animals received two intramuscular (IM) and one intranasal (IN) candidate vaccine at 3-week intervals (days 0, 21, and 51). The challenge study was performed intranasally with 5×10^6^ pfu of SARS-CoV-2 35 days post-vaccination. None of the vaccinated mice, hamsters, guinea pigs, or rabbits showed any changes in general clinical observations; body weight and food intake, clinical indicators, hematology examination, blood chemistry, and pathological examination of vital organs. Safety of vaccine after the administration of single and repeated dose was also established. Three different doses of candidate vaccine stimulated remarkable titers of neutralizing antibodies, S1, Receptor-Binding Domain (RBD), and N-terminal domain (NTD) specific IgG antibodies as well as IgA antibodies compared to placebo and control groups (P<0.01). Middle and high doses of RAZI-COV PARS vaccine significantly induced a robust and quick immune response from the third-week post-immunization. Histopathological studies on vaccinated hamsters showed that the challenge with SARS-CoV-2 did not induce any modifications in the lungs. The protection of the hamster was documented by the absence of lung pathology, the decreased virus load in the lung, rapid clearance of the virus from the lung, and strong humoral and cellular immune response. These findings confirm the immunogenicity and efficacy of the RAZI-COV PARS vaccine. Of the three tested vaccine regimens, the middle dose of the vaccine showed the best protective immune parameters. This vaccine with heterologous prime-boost vaccination method can be a good candidate to control the viral infection and its spread by stimulating central and mucosal immunity.

## Introduction

A viral outbreak of a new coronavirus infection, COVID-19, caused by the severe acute respiratory syndrome coronavirus 2 (SARS-CoV-2), emerged in China (Wuhan) in late December 2019 and rapidly spread globally (1). As of March 16, 2022, COVID-19 has infected more than 501 million people with about 6.1 million deaths worldwide (2). SARS-CoV-2 is a single-stranded, enveloped, positive-sense RNA virus that belongs to the family Coronaviridae, genus *Betacoronavirus* (3–5). The transmission rate of SARS-CoV-2 is higher than other coronaviruses infecting humans, including SARS-CoV, human coronavirus (HCoV) 229E, Middle East respiratory syndrome coronavirus (MERS-CoV), NL63, HKU1, and OC43 (6). However, the rate of case fatality by COVID-19 is lower than those reported for MERS and SARS (6). Regarding the dreadful scenario and pandemic nature of COVID-19, effective and safe vaccines appeared to be the most appropriate intervention to control the pandemic. Globally, several research teams developed efficacious and safe vaccine candidates to prevent infections caused by this virus through the induction of long-lived and protective immune responses. Vaccine development should be guided by speed manufacturing, deployment, and global access. Therefore, considerable efforts were made by the scientific and pharmaceutical community for the production of a number of vaccine candidates based on traditional to next-generation approaches, from which some vaccine formulas are under preclinical and clinical evaluation (7). These vaccines are comprised of nucleic acid-based vaccines, subunit vaccines, vector non-replicating vaccines, vector replicating vaccines, live attenuated vaccines, vaccines based on virus-like particles, and inactivated vaccines (2). According to the World Health Organization, there are 185 COVID-19 vaccine candidates under preclinical evaluation and 119 under clinical studies in humans until 10 March 2022, comprising of protein viral vector, subunit, inactivated vaccines, RNA or DNA vaccines, live attenuated vaccines, and VLP vaccines (8). Until now, Pfizer–BioNTech, Johnson & Johnson, Moderna, AstraZeneca–University of Oxford, Sinovac Biotech Gamaleya, Sinopharm, Novavax, and Bharat Biotech have reported effective vaccines and more than half of the world population is already vaccinated against SARS-CoV-2 using currently available vaccines (7). The greatest advantage of recombinant vaccines is their potential for comparatively simple large-scale and low cost production, and decreased side effects (9–11). Furthermore, the recombinant protein vaccines represent promising candidates with relatively high safety profile, especially in the geriatric population as well as delivery requirements and storage that facilitate broader usage in developing countries without sufficient medical facilities (12, 13). In comparison with the recombinant protein vaccines, there are major drawbacks to the adenovirus-vector vaccines such as Oxford’s AstraZeneca and India’s Covishield COVID-19 vaccines that use a mild human adenovirus. Human adenoviruses circulate widely, causing the common cold, and some people harbor antibodies that will target the vaccine, making it ineffective. Moreover, from a manufacturer’s perspective, one of the drawbacks of this vaccine type is that it would require live adenovirus to be grown in large quantities in the lab first that will need precautions to be taken to avoid infecting the people involved in its production (14). On the other hand, a major downside of COVID-19 mRNA vaccines such as Pfizer/BioNTech vaccine as well as Moderna is that mRNA is easily degraded and unstable that induce strong immunogenicity, triggering unnecessary immune response (15). Furthermore, the most commonly reported side effect from the Pfizer/BioNTech vaccine has been recorded as pain around the injection site, headache, fever, fatigue, and muscle pain (16, 17). The vast majority of recombinant vaccines are based on spike proteins (S) and designed to stimulate host humoral and cellular immune responses (9, 18–20). Recently, Clover Biopharmaceuticals in collaboration with GlaxoSmithKline (GSK) produced the vaccine candidate of protein-based COVID-19 with GSK’s adjuvant system (AS03) that named Clover’s COVID-19 vaccine. Clover has improved an S-Trimer subunit of COVID-19 vaccine using the Trimer-Tag approach and an expression system of mammalian cell culture (9, 21). Another company, Vaxine Pty Ltd/Medytox, has also established a recombinant spike protein-based vaccine with the Advax™ adjuvant system (Vaxine’s COVID-19 vaccine) that showed a potent immunogenicity profile (22). Therefore, it has been shown that one of the most promising targets for a vaccine developed against SARS-CoV-2 is the viral surface spike protein (11, 23). The S protein of SARS-CoV-2 with a size of 180–200 kDa and a total length of 1273 amino acids consists of two subunits S1 (surface unit, 14–685 residues) and S2 (transmembrane unit, 686–1273 residues) with the higher immunogenicity that could efficiently elicit the production of neutralizing antibodies within the immunized system, thereby protecting them from virus infection (24). The S1 unit binds the cellular receptor through its Receptor-Binding Domain (RBD), whereas the S2 unit mediates viral fusion to cell membranes and virus entry. The activation of S protein is associated with the S1/S2 spike cleavage sites through host cell proteases (25). SARS-CoV-2 also uses a distinguishable trimeric S protein on their viral envelopes to gain entry into its host cells. The trimeric S protein of SARS-CoV-2 binds with high affinity to the human cell surface receptor, angiotensin-converting enzyme 2 (ACE2), and regulates subsequent viral entry *via* membrane fusion (26). Trimeric S protein preserves the native trimeric form of S protein in the prefusion structure of the antigenic epitope, which is crucial for viral neutralization (27). Recent investigations emphasized the immunization properties of the recombinant spike protein in mice that effectively protect them from SARS-CoV-2 infection (11, 28–30). Some recombinant protein vaccines have already proven their efficiency and safety in the prevention of viral diseases like hepatitis B (18). Therefore, in this study, we designed a recombinant-based vaccine in which the subunits of S1, S2, and open-state S-Trimer proteins of SARS-CoV-2 are encoded into the mammalian expression vector PK001 for the production of recombinant proteins. The resulting vaccine candidate was assessed for its ability to elicit cellular and humoral immune responses in four experimental animal species. Furthermore, the ability of vaccine in stimulation of IgA antibodies levels were assessed for 210 days. Virus challenge experiments were conducted on immunized hamsters to define the efficacy and protective effect of the vaccine against SARS-CoV-2.

## Materials and Methods

### Ethics Statement

All procedures involving animals were approved by the Institutional Biosafety Committee and Institutional Animal Ethics Committee of the Razi Vaccine and Serum Research Institute (RVSRI), Karaj, Iran (IR.RVSRI.REC.1399.001). All experiments complied with the guidelines laid down by the Committee and all relevant ethical regulations. The assays were performed in a facility of biosafety level 3 (BSL3) in RVSRI. All experiments were carried out in agreement with the ARRIVE guideline (31) and regulated by the Committee on *Animal Research* and *Ethics* (CARE) of the RVSRI.

### Rodent Models

In the study, 575 SARS-CoV-2-seronegative rodents (6–8-weeks-old equal female/male Syrian hamster, 6–8-weeks-old equal female/male BALB/c mice, and 6–8-weeks-old equal female/male Pirbright guinea pig) and 12–15-weeks-old equal female/male NZW rabbits in four groups were supplied from Razi vaccine and serum research institute (Karaj, Iran). A different group of each animal was divided into five groups and received spike protein subunits S1, S2, and S-Trimer of SARS-CoV-2 formulated in adjuvant systems of Razi Adjuvant System 01 (RAS-01). One group as placebo received the adjuvant RAS-01 alone (placebo, group A), another group as control received the phosphate-buffered saline (PBS) (control, group B), and three groups immunized intramuscularly (IM) with a low dose of candidate vaccine (group C), middle dose of candidate vaccine (group D), and high dose of candidate vaccine (group E) at 0 and 21days. All animals received one more booster dose of intranasal (IN) candidate vaccine on day 51. The number of animals in each group for safety evaluation was 50 BALB/c mice, 10 hamsters, 10 guinea pigs, and five rabbits. Furthermore, the number of animals in each group for immunogenicity evaluation were 15 BALB/c mice, 10 hamsters, 10 guinea pigs, and five rabbits. Syrian hamsters are highly sensitive to SARS-CoV-2 and provide a reliable animal model for vaccine evaluation against the virus (32).

### *In Vitro* Culture of SARS-CoV-2

The growth of African green monkey kidney clone E6 cells (Vero E6, ATCC^®^ CRL-1586™) were performed under the medium of Dulbecco’s Modified Eagle’s Medium (DMEM) comprised of nonessential amino acids (NEAA), 2 mM L-glutamine, 10% fetal bovine serum (FBS), 12.5 Units/ml nystatin (P/S/N), 100 Units/ml penicillin, and 0.1 mg/ml streptomycin (GIBCO, USA). Cells were cultured with 95% humidity and 5% CO2 at 37°C. SARS-CoV-2 was isolated from clinical human specimens in this study by RVSRI, Karaj, Iran (GISAID accession EPI_ISL_1398937). The propagation and titration of the virus were performed on Vero E6 cells. A BSL3 facility was used for working and handling the Iranian SARS-CoV-2 in agreement with the biosafety guidelines of the RVSRI.

### Vaccine Design and Formulation

In this study, S1, S2, and open-state S-Trimer of SARS-CoV-2 were selected for the construction of vaccine. The DNA constructions of SARS-CoV-2 derived spike proteins were designed based on Genbank accession number MN908947. The recombinant S1 and S2 subunit proteins contain the amino acids 1-674 and 685-1211, respectively. The S1 and S2 express with a C-terminal human IgG Fc-tag, while the Fc tag were removed and replaced by a T4 folded sequence in S-Trimer design. The DNA for S1 and S2 antigens was codon-optimized for Expi293F human cells (Cat#A14527), according to their manufacturer recommendations. The gene related to open-state S-Trimer antigen was codon-optimized to express in ExpiCHO-S™ Cells (Cat # A37785). The expression vectors encoding S1/S2 antigens were used to transiently transfect Expi293F cells using polyethylenimine. The S1 and S2 proteins and open-state S-Trimer were purified from filtered cell supernatants using Pierce Protein A resin (Thermo-Fisher, Dreieich, Germany). Plasmid encoding the S-Trimer antigen was transiently transfected into ExpiCHO-S™ Cells using polyethylenimine. S-Trimer proteins were purified from cell supernatants by Superose 6 10/300 column (Cytiva, Marlborough, USA). The recombinant S1 and S2 proteins and open-state S-Trimer proteins were approved by mass spectroscopy, western blots, and SDS-PAGE gels as described previously (33). After purification of the desired recombinant antigens, a cocktail of these antigens with the same proportion (S1, S2, and open-state S-Trimer) was prepared. and the recombinant proteins with a 1:1 ratio were mixed with the adjuvant and the mixture was stored at 4°C. The physicochemical properties of adjuvant are investigated and controlled by DLS (Malvern, France). The same process was used in the production of inhaled RAZI-COV PARS. Razi Adjuvant System-01 (RAS-01) is an oil-in-water emulsion comprised of sesame, olive, and soybean oils (ADM, France) as well as the non-ionic surfactant Tween 80 (Merck Germany) with ratios (v/v) of 10:10:10:5. To obtain equilibrium, the prepared RAS-01 was stirred for 30 min. The preparations were homogenized by magnetic stirrer 7000 rpm, followed by sonication (Hielscher, Germany) for 30 min at 50°C operating at 100 KHz to obtain Nano emulsion (NEs). The last product was passed through a microfluidizer device to make the emulsion completely homogenous. The size and zeta potential for the stability of droplets were evaluated by dynamic light scattering (DLS) through the Malvern zeta analyzer (France). Droplet sizes of the NEs were measured with a range of 8-6500 nm and a range of 20–200 mV, respectively.

### Safety Evaluation

The safety evaluation of candidate vaccine, including the toxicity, toxicity following repeated doses, and pyrogenicity assay, was performed according to ICH M3 instructions (International Council for Harmonisation of Technical Requirements for Pharmaceuticals for Human Use) on non-clinical safety studies (34). Syrian hamsters, BALB/c mice, and guinea pigs were used to evaluate the safety and immunogenicity, while NZW rabbits were also used for safety evaluation. Throughout the study, blood samples were collected in either fasting or non-fasting condition at variable time points. Hematology measurements include hematocrit (Hct), hemoglobin concentration (Hb), erythrocyte count, total and differential leukocyte counts, mean corpuscular hemoglobin (MCH), and mean corpuscular volume (MCV). Clotting time (CT), prothrombin time (PT), prothrombin time (PTT), and platelet count (PLT) were measured to assess likely clotting concerns. The evaluation of the hematopoietic system through the reticulocyte counts, the liver function through the levels of ALT and AST, the kidney and cardiac function tests through the analysis of lactate dehydrogenase (LDH) and creatine phosphokinase (CPK) were also performed. Further, the indicators of cellular function such as albumin, calcium, chloride, cholesterol (total) which may indicate liver toxicity, creatinine, blood glucose which may possibly show renal toxicity, phosphorus, globulin (calculated), potassium, protein (total), triglycerides (fasting), sodium, and urea nitrogen were measured. Urine analyses including the determination of urine volume, specific gravity, pH, glucose, and protein along with microscopic evaluation for sediment and presence of blood/blood cells were performed. Further, vital organs such as the brain, heart, kidneys, liver, lungs, spleen, gastrointestinal tract, and lymph nodes were dissected for subsequent histopathological examination. After toxicity analysis, the maximum recommended starting dose (MRSD) based upon no observed adverse effect level (NOAEL) in each species was determined.

### Vaccine Dose-Escalation and Immunogenicity Studies

Escalating dose-finding trials of RAZI-COV PARS were performed according to recently described protocols (10). Increasing doses of recombinant proteins combined with adjuvant of RAS-01 were administrated in heterologous vaccine regimens in different animals, i.e., BALB/c mice: [0.5µg (low), 1 µg (middle), and 2 µg(high)]; hamster: [1µg(low), 2µg (middle), and 3 µg(high)]; guinea pig: [2µg(low), 4 µg(middle), and 5 µg(high)]; and rabbit: [4µg(low), 6 µg(middle), and 8 µg (high)]. All groups, except mice, received three doses (two IM and one IN) of candidate vaccine or adjuvant at 3-week intervals (days 0, 21, and 51). The mice’s airway is very small and we were not able to use the spray for IN dose (35). Blood samples were collected on days 0, 14, 35, 65, 120, 150, and 180 post-immunizations for assessment of specific antibodies and neutralizing antibody titers. However, for evaluation of IgA antibodies, mucosal and blood sampling was continued until day 210. Animals were monitored daily for injection site reactions and clinical signs. The monitoring of rectal temperature was performed every 24 hours up to 3 days’ post-immunization. Body weight was checked every 24 hours for 36 weeks.

### Enzyme-Linked Immunosorbent Assays (ELISA)

The S1, RBD, and NTD specific IgG antibody titer of collected serum samples from all immunized animals were evaluated through in-house ELISA. Briefly, 96-well high-binding plates (Greiner, Austria) were coated with 100ngr/well of S, RBD, and NTD antigens diluted in sterile carbonate buffer at 4°C overnight. Following standard blocking with 3% of skimmed milk for 2 hrs at 37°C and subsequent washing, the plates were incubated with vaccinated mice, guinea pigs, hamsters, and rabbit’s sera at a dilution of 1:50 for 1 hr at 37°C in duplicates. After washing three times, anti-mouse IgG-HRP conjugates, anti-guinea pig IgG-HRP conjugates, anti-hamster IgG-HRP conjugates, and anti-rabbit IgG -HRP conjugates were diluted 1/15000, 1/80000, 1/10000, and 1/40000 for mice, guinea pig, hamster, and rabbit, respectively, and were used as secondary antibodies (Sigma, USA) followed by washing and detection with 3,5,3′,5′-tetramethylbenzidine (Millipore, USA).

### Evaluating the IgG Subtype (IgG1 and IgG2a) in Immunized Mice Sera

After confirmation of the presence of specific IgG antibodies, the analysis of IgG2a and IgG1 isotypes (markers for Th1 and Th2 lymphocytes, respectively) was carried out by Mouse Immunoglobulin Isotyping Recombinant Rabbit Monoclonal Anti-Mouse IgG1, IgG2a (Abcam, cat. #ab125904) based on the manufacturer’s instructions (Abcam, Cambridge, UK). All sera were tested in duplicate and sample values were assessed in comparison with the control’s absorbance values.

### Human ACE2 Protein (hACE-2) Binding Assay

For the ACE2 binding assay, the human ACE2 protein (hACE-2) (Native Antigen UK) was coated overnight and blocked 3 hrs with 0.1% BSA. Then, His tag RBD in different concentrations was added to mouse sera with 1/100 dilution and the samples were incubated for 2 hrs at 37°C. After incubation, centrifuged 6000 RPM and supernatant were removed from tube and added in 96-well ELISA plate and incubated for 2 hrs at 37°C. After that blocking buffer was removed and washed by 200 μL 1X washing buffer once. In next step, 100 μL of 1500X HRP-conjugated Anti-His tag antibodies were added to the 96-well plates and incubated for 1h at 37°C. Finally, the secondary antibody was removed and the 96-well plates were washed by 200 μL using PBST for four times. Then, 100 μL TMB reagents were added to each well. After 15 min, 100 μL of stop solution was added to the wells and read the absorbance (OD450) of each well with an ELISA plate reader.

### ELISA Assays for Assessment of RBD Specific IgA Antibody in Saliva and Serum Samples

Hamsters were used to investigate the level of IgA antibodies against RBD in the upper respiratory tract and serum at regular intervals as described previously elsewhere (36). Saliva and serum samples were treated with Triton^®^ X-100 for inactivation of the virus. Following inactivation, saliva and serum samples were stored in -80°C overnight. Then, the saliva samples were centrifuged at 15,000 rpm for 10 min in 4°C; the supernatant was then immediately transferred to a clean tube and allocated. The samples were examined for the levels of RBD-specific IgA antibody on days 0, 14, 28, 35, 60, 120, 150, 180, and 210 after the first (IM), second (IM), and third dose (IN) of the candidate vaccine by ELISA. Briefly, 100 ng/well of RBD in Carbonate-Bicarbonate buffer with pH 9.6 were coated on 96-well ELISA plates (Greiner) and incubated overnight for 24 hrs at 4°C. Plates were washed once and blocked with 200 μL/well of 3% skimmed milk (Sigma, USA) for 3 hrs at 37°C. After washing by PBS, the plates were incubated with 100 μL/well samples obtained from hamsters of control group, immunized with vaccination for 2 hrs at 37°C. The wells were then washed three times by PBS. Following the washing step, incubation was performed with 100 μL/well of Anti Quin pig IgA HRP (Sigma, USA) diluted to 1:5000 in 3% skimmed milk and incubated at 37°C for 2 hrs. In the end, plates were washed four times with PBS-T (0.05% Tween 20 in PBS) and then tetramethyl benzidine (TMB) (Sigma, USA) was added. Duplicate analyses were performed on each sample.

### Virus Neutralizing Test (VNT)

The VNT analysis was performed to evaluate the protective values of RAZI-COV PARS candidate vaccine. SARS-CoV-2 (GISAID accession EPI_ISL_1398937) was isolated from clinical human specimens in this study. The titration of virus was performed from 1 log to 11 log (in serial 1 log dilutions) to obtain a 50% tissue culture infective dose (TCID50) on 96-well culture plates of VERO cells. Serum samples obtained from all animals were heat-inactivated at 56°C for 30 min. Two-fold serial dilutions of serum samples with the starting dilution of 1:4 (i.e., 1/4, 1/8, 1/16, 1/32, 1/64) were then mixed with an equal volume of 100 TCID50 of the SARS-CoV-2 and incubated at 37°C for 1 hr under 5% CO2. The pre-incubated SARS-CoV-2 was then added to 100 µL of the VERO cells (4 X10^5^ cells/ml) in duplicate and incubated for 10 days at 37°C under 5% CO2. After the incubation, an inverted optical microscope was used to enumerate the formation of cytopathic effect (CPE) in wells. The highest serum dilution that protected more than 50% of the cells from CPE was taken as the neutralization titer (37).

### Evaluating the Cellular Immune Response by CFSE Proliferation Assay

Cell suspensions of spleen from vaccinated BALB/c mice were evaluated for cell proliferative ability of splenic mononuclear cell (MNCs), cytokine production, and surface marker expression. Briefly, cell suspensions were prepared from recently sacrificed animals and were cultured in medium containing 10 ml of RPMI medium (Gibco, USA) with 5% fetal bovine serum for 5 hrs at 37°C. Then cells were washed with PBS supplemented with 2% fetal bovine serum (FBS). Red blood cells were depleted from cell suspension by RBC Lysis solution for 10 min at room temperature. The process was followed by threewashes with PBS/2% FBS. Then, cells were cultured within RPMI medium (Gibco, USA), containing penicillin/streptomycin 1%, and 10% FBS. The proliferative ability of MNCs of spleen from BALB/c mice was measured by Carboxyfluorescein succinimidyl ester (CFSE) (BioLegend, USA) proliferation assay on day 35 of vaccination procedure using a flow cytometer (BD FACS Lyric, USA) (38, 39). For this purpose, CFSE labeling performed on separated MNCs and the tube containing the mixture of cells (1×10^6^ cells in 1 ml PBS/2% FBS) and 1 µL of 5 mM CFSE (the final concentration of 1 µg/ml) were rapidly inverted and vortexed for 10 sec. After 15 min incubation in 37°C and two times washing, the CFSE stained MNCs were seeded in 96-well plates (1×10^5^ cells in 100 µl of RPMI with 10% FBS). The cells stimulated either with 5 µl of PHA (GIBCO, USA), or spike SARS-COV-2 proteins (0.3 µg/ml) and heat-inactivated SARS-COV-2 viruses. Then incubated in 37°C with 5% CO2 for 72 hrs until analysis using a flow cytometer (BD FACS Lyric, USA) and FACSuite V1.2.1software.

### Evaluating the Cell Markers and Intracellular IFN-γ of MNCs in Vaccinated Mice

MNCs collected from mice spleen were cultured as described above. After 48 hrs of incubation with PHA, S1, and S2 proteins (0.3 µg/ml) and inactivated SARS-COV-2 viruses, the cells were analyzed for cell surface markers and intracellular IFN-γ cytokine. Cell surface staining was performed with APC-Cy7 conjugated anti-CD3 (BD, 560590), PerCP-Cy5.5 anti-CD4 (BD, 550954), APC-R700 anti-CD8 (BD, 561093), and phycoerythrin-Cy 7 (PE- Cy7) anti-CD27 (BD Pharmingen, 555275), APC anti-CD62L (BD, 104505), and Alexa flour 488 (Ax-488) anti-CD127. Therefore, cells at the density of 10^6^/100 μL in staining buffer were incubated with desired conjugated antibodies for 30 min at 4°C in the dark. Cells were then washed twice using PBS/2% FBS and centrifuged at 500 g for 5 min. For intracellular IFN-γ cytokine staining, Golgi Plug (BD, 2301kz) treated cells fixed with 250 µl of paraformaldehyde (PFA) (BD Cell fix) for 20 min at RT. The cells permeabilized with 0.2% tween 20/PBS for 15 min at room temperature (RT). Cells were stained with PE- anti IFN-γ antibody (BD Pharmingen, 555275) in 100 μL of 0.1% tween 20 in PBS/FBS 2% (perm/wash buffer) for 30 min at 4°C, then washed twice with wash buffer and data acquisition were performed by BD FACS Lyric flow cytometer and analyzed by FACSuite V 1.2.1 software.

### Evaluation of Cytokine Level by ELISA

The level of the cytokines such as TNF-α, IFN-γ, IL-2, IL-4, IL-6, IL-17 in the supernatants of MNCs cultured specifically stimulated with S antigens were measured by commercial ELISA kits (R&D System, USA) according to the manufacturer’s instructions. The OD value (450 nm) was read by Cytation 5 imaging reader (BioTek, USA). The results are expressed as pg/ml.

### SARS-CoV-2 Challenge Study in Hamsters

The immunized hamsters (103 ± 20g) with middle dose of vaccine, control, and placebo groups were anesthetized and challenged intranasally with 5×10^6^ pfu of SARS-CoV-2 (GISAID accession EPI_ISL_1398937, Karaj, Iran) on 35 days’ post vaccination (14 days after the second dose). Four challenged hamsters from each group were euthanized on days 3, 5, and 7 post challenges to collect lung samples for histopathology, viral RNA estimation, and titration analysis. Lung samples of hamsters were collected in virus transport media (1 ml) on every alternate day post inoculation for viral load estimation. Furthermore, the weights of Syrian hamsters were recorded daily for 7 days after SARS-CoV-2 challenge.

### RNA Isolation and Real-Time Polymerase Chain Reaction (RT-PCR)

Total RNA was extracted at 35th day of vaccination from mouse splenocytes by using RNA extraction kit (QIAGEN RNA extraction kits, German). The quantity and quality of extracted RNA were tested by spectrophotometric and gel electrophoresis analysis, respectively. Subsequent to DNase I treatment (Fermentas, USA), the synthesis of complementary DNA (cDNA) was performed from 1mg of total RNA through reverse transcriptase (RevertAid™, Thermo Scientific, Lithuania) with an oligo (dT) primer through the manufacturer’s instructions. Real-time PCR was performed to evaluate the expression of selected genes including GAPDH, β-actin, IL-1, IL-2, IL-4, IL-10, IL-12p40, IL-13, IL-17, IL-18, TNF-α, INF-γ, and TGF through an iCycler (ABI 7300 Real-Time PCR System (Applied Biosystems, Austin, USA). The thermal programs of first denaturation were 3 min at 95°C and then 40 cycles of denaturation at 20 sec at 95°C, annealing for 20 sec at 52.5°C and extension for 20 sec at 72°C. The triplicate reaction was performed for each sample. The mixture reaction consisted of 12 µl of TaKaRa SYBR Green dye, PCR master mix, 500 nM from each forward and reverse primer, 1 µl cDNA, and 1 µl DEPC water (Takara Bio TB Green Premix Ex Taq (cat. # RR820A). The GAPDH gene was applied as a reference gene for normalization of the target gene expression for each mRNA. After verification of primers efficiencies, gene expression was determined according to the ΔΔCt using IQ5 software (BIO-RAD).

### Statistical Data Analysis

Data analyzing geometric mean and SD were calculated based at different time points. Seroconversion as measured by ELISA was defined as an increase by a factor of 4-fold or more in antibody area under the curve (AUC) over baseline. Analysis of variance (ANOVA) was used for statistical comparisons of IgG antibodies among different groups and different animals, the cellular immune response and the ACE2 binding inhibition assays with different doses of candidate vaccines. For all statistical tests α = 5% was considered as significant level. All analyses were performed using Stata software ver.14, and all graphs were generated using GraphPad Prism (version 8.3) software. One-way ANOVA, T test, and Mann-Whitney test was used to compare the follow-up criteria between the animal groups. *p*-values are reported as follow: *P*>0.05 as statistically not significant, *P*<0.05 as statistically significant, *P*<0.01 as statistically very significant, and *P*<0.001 as statistically extremely significant.

## Results

### Production, Characterization, and Analysis of SARS-CoV-2 S1, S2, and Open-State S-Trimer Proteins

**Figure 1:**
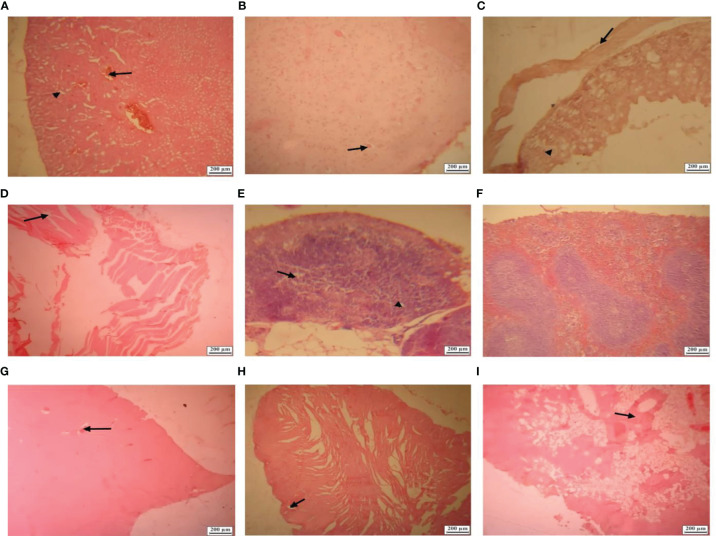
Histological lesions in BALB/c mice tissues in high toxic dose of candidate vaccine (20µg). **(A)** Section from a kidney showing hyperemia (arrow), intratubular edema and multiple foci of edema (arrowhead) in kidney parenchyma (Hematoxylin and eosin×100). **(B)** Section from a brain showing small foci of perineuronal edema (arrow) (Hematoxylin and eosin×100). **(C)** Section from an intestine showing villous atrophy (arrowhead), submucosal edema (arrow) and vacuolated enterocytes (Hematoxylin and eosin×100). **(D)** Section from an injection site showing edema between muscles bundles (arrowhead) (Hematoxylin and eosin×100). **(E)** Section from a mediastinal lymph node showing lymphatic depletion (lymphoid reduction), decreased paracortical lymphocytes (arrow) and small foci of edema (arrowhead) (Hematoxylin and eosin×100). **(F)** Section from a spleen showing no lesion (Hematoxylin and eosin×100). **(G)** Section from liver showing foci of hyperemia (arrow) in the sinusoids and parenchymal edema with mild fatty degeneration (Hematoxylin and eosin×40). **(H)** Section from heart showing mild hyperemia between cardiac muscle fibers (arrow) (Hematoxylin and eosin×40). **(I)** Section from lung showing parenchymal and vascular sections hyperemia as well as serous secretions (arrow) and increasing in alveolar septa (Hematoxylin and eosin×40).

**Figure 2:**
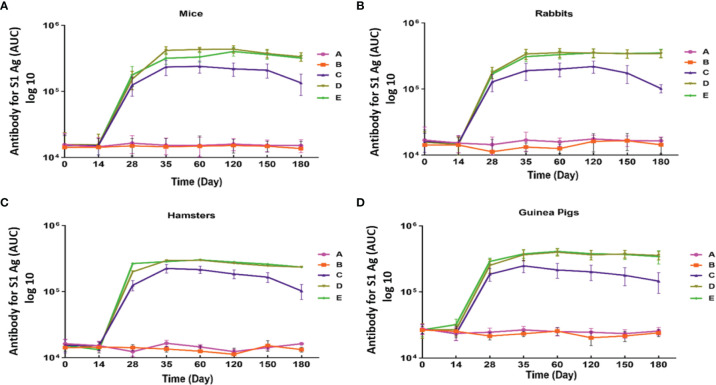
Anti-SARS-CoV-2 immunoglobulin-G (IgG) antibody titers against S1 antigen in different animal models immunized with candidate vaccine. **(A)** Anti-SARS-CoV-2 immunoglobulin-G (IgG) antibody titers against S antigen for 180 days from the day it is received candidate vaccine in mice. **(B)** Anti-SARS-CoV-2 immunoglobulin-G (IgG) antibody titers against S1 antigen for 180 days from the day it is received candidate vaccine in rabbit. **(C)** Anti-SARS-CoV-2 immunoglobulin-G (IgG) antibody titers against S1 antigen from 1st to 180 day of immunization with candidate vaccine in hamster. **(D)** Anti-SARS-CoV-2 immunoglobulin-G (IgG) antibody titers against S antigen for 180 days from the day it is received candidate vaccine in in Pirbright guinea pig. Data are presented as mean ± standard deviation (SD). The levels of statistical significance for differences between test groups were determined using one-way ANOVA followed by Tukey’s *post hoc* test. Statistical comparison was done by comparing the vaccinated group with the placebo group as a control. All animal groups receiving candidate vaccine (low, middle, and high dose) showed a statistically significant difference of IgG antibody in compared with the groups receiving placebo and adjuvant (P<0.001) on days 28, 35, 120 and 180. Placebo (adjutant of RAS-01) (group A), phosphate buffered saline (PBS, group B), low dose candidate vaccine (group C), middle dose candidate vaccine (group D), and high dose candidate vaccine (group E).

The S1 and S2 subunit recombinant proteins were fused to Fc fragment of human IgG in order to extend the serum half-life. They were ligated with the PK001 mammalian vector and expressed by human Expi293F cell line. S1-Fc and S2-Fc fusion proteins were purified from the culture supernatant using a Protein-A resin column. The purity of S1-Fc and S2-Fc proteins was investigated by running SDS-PAGE gel under non-reduced and reduced conditions. At first inspection by SDS-PAGE gel and coomassie blue staining, a band with molecule weight of 130 kD was identified as the S1-Fc, while a band of 90 kD appeared as S2-Fc and 120 kD for open-state S-Trimer as depicted in **Figure 1S**. The Western blot analysis using the anti-S full antibody as a primary and anti-human IgG HRP (Sigma USA) as a secondary antibody also confirmed the robust expression of S1, S2, and open-state S-Trimer recombinant proteins (**Figure 1S**). HPSEC chromatogram of purified recombinant protein of S1, S2, and S-Trimer showed purity of 95% and qualified for formulation as a vaccine product (**Figure 1S**). Our analysis showed that the size of the vaccine particle is 200 to 500 nm and the particle charge is negative (**Figure 2S**).

### Safety Evaluation of RAZI-COV PARS Candidate Vaccine

No clinical manifestations such as weight loss, fever, or local complications like redness, swelling, or inflammation were observed in any of the experimental or control groups. In terms of hematology and blood indices, no increase or suppression of the target cells was seen in BALB/c mice, Syrian hamsters, guinea pigs, or NZW rabbits (**Table 1**). Abnormal blood cells were also not observed. There were no significant electrolyte changes in biochemical blood tests showing normal ranges throughout the evaluation period. Liver function tests (LFT) were also followed up normally in all experimental groups. The coagulation tests including PT and PTT were also normal. Renal function evaluation tests did not change significantly throughout the evaluation period and were reported as normal. No adverse events or mortality were observed during the evaluation period. Based on clinical, hematological, biochemical, or coagulation studies in BALB/c mice, Syrian hamsters, Pirbright guinea pig, and NZW rabbits as well as its histopathological results with different doses showed that the vaccine candidates in low, middle, and high doses as well as the adjuvant alone are safe. In the gross evaluation of animals receiving high, middle, and low doses of candidate vaccine, none of the carcasses had any abnormal appearance. The histopathological analysis also showed that the selected tissues including kidney, heart, lymph nodes, intestines, spleen, lung, liver, central nervous system, and injection site did not indicate reportable pathognomonic changes in the group receiving the low, middle, and high dose of candidate vaccine [0.5µg (low), 1 µg (middle), and 2 µg(high)], despite the presence of minor transudate edema in kidney parenchyma, brain, and intestine of mice receiving a highly toxic dose of candidate vaccine (20µg) in safety test (**Figure 1**).

**Table 1:** The detail of animal studies for evaluation of safety pharmacology, including toxicity, toxicity upon repeated doses and pyrogenicity assessment, performed in accordance with the ICH M3 guidelines.

Animal species/strain	BALB/c mice	Golden hamster	Guinea pigs (pirbright)	New Zeeland white rabbit
Number of animals	50/group	10/group	10/group	5/group
Administration	2 IM in 3w interval	2 IM (in 3w interval) + 1 IN (4w after 2nd dose)	2 IM (in 3w interval) + 1 IN (4w after 2nd dose)	2 IM (in 3w interval) + 1 IN (4w after 2nd dose)
Dose levels for safety	5µg/10µg/20µg	5µg/10µg/20µg	5µg/10µg/20µg	5µg/10µg/20µg
Clinical observations	Once daily for the first 3 days then twice a week	Once daily for the first 3 days then twice a week	Once daily for the first 3 days then twice a week	Once daily for the first 3 days then twice a week
Body weight	Once daily for the first 3 days then at least weekly	Once daily for the first 3 days then at least weekly	Once daily for the first 3 days then at least weekly	Once daily for the first 3 days then at least weekly
Body temperature	Continuously recorded during the whole experiment, Once daily for the first 3 days then weekly	Continuously recorded during the whole experiment, Once daily for the first 3 days then weekly	Continuously recorded during the whole experiment, Once daily for the first 3 days then weekly	Continuously recorded during the whole experiment, Once daily for the first 3 days then weekly
Injection site reaction (local tolerance)	Macroscopic (once daily for the first 3 days then at least weekly/Microscopic (histopathology) examination	Macroscopic (once daily for the first 3 days then at least weekly/Microscopic (histopathology) examination	Macroscopic (once daily for the first 3 days then at least weekly/Microscopic (histopathology) examination	Macroscopic (once daily for the first 3 days then at least weekly/Microscopic (histopathology) examination
Laboratory investigations	Haematology, blood biochemistry, serology and urinalysis	Haematology, blood biochemistry, serology and urinalysis	Haematology, blood biochemistry, serology and urinalysis	Haematology, blood biochemistry, serology and urinalysis
Post-mortem organs investigations	LNs, liver, respiratory tract, GI tract, nervous system, spleen, kidney, heart	LNs, liver, respiratory tract, GI tract, nervous system, spleen, kidney, heart	LNs, liver, respiratory tract, GI tract, nervous system, spleen, kidney, heart	LNs, liver, respiratory tract, GI tract, nervous system, spleen, kidney, heart

### RAZI-COV PARS Candidate Vaccine Elicits S1-, RBD-, and NTD-Specific IgG Antibody Responses in Animal Models

**Figure 3:**
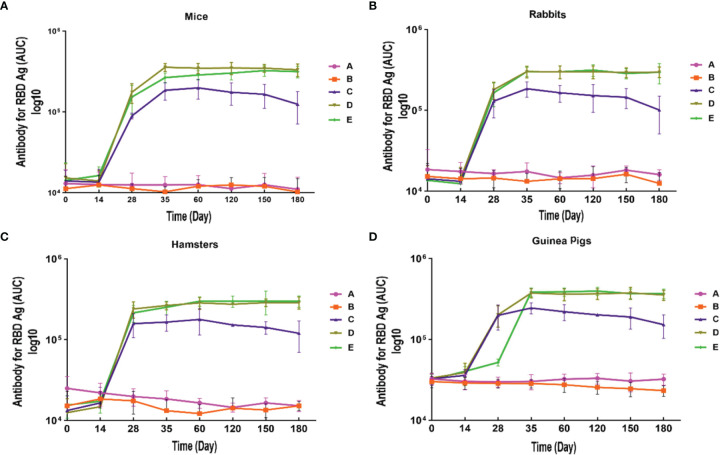
Anti-SARS-CoV-2 immunoglobulin-G (IgG) antibody titers against RBD antigen in different animal models immunized with candidate vaccine. **(A)** Anti-SARS-CoV-2 immunoglobulin-G (IgG) antibody titers against RBD for 180 days from the day it is received candidate vaccine in mice. **(B)** Anti-SARS-CoV-2 immunoglobulin-G (IgG) antibody titers against RBD for 180 days from the day it is received candidate vaccine in rabbit. **(C)** Anti-SARS-CoV-2 immunoglobulin-G (IgG) antibody titers against RBD for 180 days from the day it is received candidate vaccine in hamster. **(D)** Anti-SARS-CoV-2 immunoglobulin-G (IgG) antibody titers against RBD for 180 days from the day it is received candidate vaccine in guinea pig. Data are presented as mean ± SD. The levels of statistical significance for differences between test groups were determined using one-way ANOVA followed by Tukey’s *post hoc* test. Statistical comparison was done by comparing the vaccinated group with the placebo group as a control. A significant increase of IgG against the RBD was observed one week after the second dose of the candidate vaccine (day 28) in all animal groups receiving the candidate vaccine and remained significantly higher until day 180 in compared with the groups receiving placebo and adjuvant (P<0.001). Placebo (adjutant of RAS-01) (group A), phosphate buffered saline (PBS, group B), low dose candidate vaccine (group C), medium dose candidate vaccine (group D), and high dose candidate vaccine (group E).

**Figure 4:**
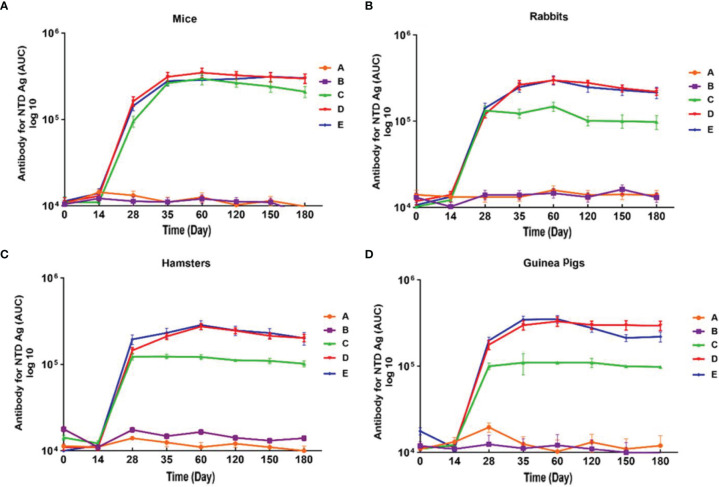
Anti-SARS-CoV-2 immunoglobulin-G (IgG) antibody titers against NTD antigen in different animal models immunized with candidate vaccine. **(A)** Anti-SARS-CoV-2 immunoglobulin-G (IgG) antibody titers against NTD for 180 days from the day it is received candidate vaccine in mice. **(B)** Anti-SARS-CoV-2 immunoglobulin-G (IgG) Antibody titers against NTD for 180 days from the day it is received candidate vaccine in rabbit. **(C)** Anti-SARS-CoV-2 immunoglobulin-G (IgG) antibody titers against NTD for 180 days from the day it is received candidate vaccine in hamster. **(D)** Anti-SARS-CoV-2 immunoglobulin-G (IgG) antibody titers against NTD in for 180 days from the day it is received candidate vaccine in guinea pig. Data are presented as mean ± SD. The levels of statistical significance for differences between test groups were determined using one-way ANOVA followed by Tukey’s *post hoc* test. Statistical comparison was done by comparing the vaccinated group with the placebo group as a control. A significant increase of IgG against the NTD was observed one week after the second dose of the candidate vaccine (day 28) in all animal groups receiving the low, middle, and high doses of candidate vaccine and remained significantly higher until day 180 in compared with the groups receiving placebo and adjuvant (P<0.001). Placebo (adjutant of RAS-01) (group A), phosphate buffered saline (PBS, group B), low dose candidate vaccine (group C), medium dose candidate vaccine (group D), and high dose candidate vaccine (group E).

IgG antibody against S1 antigen was evaluated in sera collected from immunized Syrian hamster, BALB/c mice, Pirbright guinea pig, and NZW rabbit on days 0, 14, 28, 35, 60, 120, 150, and 180. Statistical analysis of serum IgG levels produced against S1 antigen at 14 days after the first doses of candidate vaccine in all immunized animals showed no significant difference compared with day zero (p>0.05). However, significantly elevated IgG antibody responses against S1 antigen were observed one week after the second immunization (day 28) in Syrian hamsters, BALB/c mice, Pirbright guinea pig, and NZW rabbit (P<0.001) (**Figure 2**). By contrast, the sera from control animals treated with adjutant of RAS-01 (group A) and PBS (group B) showed only background-level antibody responses. The increase in anti-S1 IgG antibody was detectable after the second dose of the candidate vaccine from day 28 and continued until day 120. In groups of middle and high dose of candidate vaccine, 100% of animals showed a significant increase in anti-S1 IgG antibody from day 35 onwards (P<0.001). From day 120 to 180, the amount of anti-S IgG antibody showed a non-significant decrease in 100% of animals, but it was still significantly higher than the control (PBS) and placebo (adjutant of RAS-01) groups. The sera from all animals receiving high- and middle-dose of candidate vaccine showed no statistical significance (P> 0.05) on days 35, 120, and 180. IgG antibodies against RBD antigen were evaluated in the immunized animal’s sera on days 0, 14, 28, 35, 60, 120, 150, and 180. Statistical analysis of serum IgG levels produced against RBD at 14 days after the first dose of the candidate vaccine showed a slight increase in Syrian hamster and Pirbright guinea pig in all groups of receiving candidate vaccine and in BALB/c mice in the group receiving high dose of candidate vaccine but there were no statistically significant differences (p>0.05). However, NZW rabbit showed a slight non-significant decrease compared to day zero (p>0.05). On the other hand, a significant increase of IgG against the RBD and NTD was observed one week after the second dose of the candidate vaccine (day 28) in all animal groups receiving the candidate vaccine (P<0.001). The increase of anti-RBD IgG antibody and anti-NTD IgG antibody was significantly detectable from day 28 after immunization and continued until day 120 (p<0.01). In all immunized groups with different doses of candidate vaccine, 100% of animals showed a significant increase in antibodies against the RBD and NTD from day 35 onwards (**Figure 3**) (p<0.01). However, from day 120 to 180, the amount of IgG antibody against RBD and NTD showed a non-significant decrease in 100% of tested mice (p>0.05), but it was still significantly higher than the control (PBS) and placebo (adjutant of RAS-01) groups (P>0.05). Furthermore, the IgG antibody levels were dose-dependent, and could be stimulated with a low dose of the candidate vaccine from 28 days after administration (**Figures 2**-**4**).

### RAZI-COV PARS Candidate Vaccine Induced RBD Specific IgA Antibody Response

**Figure 5:**
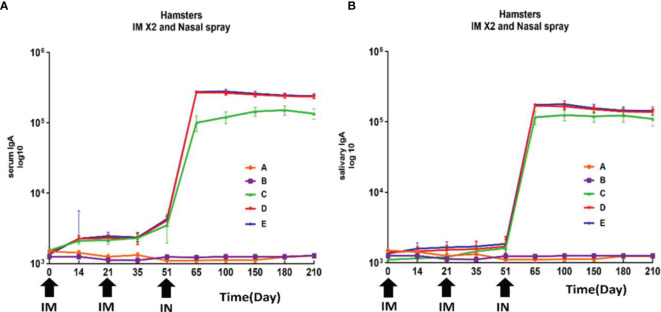
Anti-SARS-CoV-2 immunoglobulin-A (IgA) antibody titers against RBD in hamster immunized with candidate vaccine. All vaccinated animals receiving two intramuscular (IM) and one intranasal (IN) of candidate vaccine at 3-week intervals (days 0, 21 and 51). **(A)** Anti-SARS-CoV-2 immunoglobulin-A (IgA) antibody titers against RBD in serum of hamster for 210 days from the day it is received candidate vaccine; The increased IgA antibody of serum against RBD was significantly higher in animals receiving different doses of candidate vaccine until day 210 (P<0.001). **(B)** Anti-SARS-CoV-2 immunoglobulin-A (IgA) antibody titers against RBD in salivary of hamster for 210 days from the day it is received candidate vaccine. The results of IgA analysis in saliva showed a significant increase in IgA antibody level after the third dose of the candidate vaccine (IN) in hamsters (P<0.001). *P values* lower that 0.05 were considered to be statistically significant. Data are presented as mean ± SD. The levels of statistical significance for differences between test groups were determined using one-way ANOVA followed by Tukey’s *post hoc* test. Statistical comparison was done by comparing the vaccinated group with the placebo group as a control. Placebo (adjutant of RAS-01) (group A), phosphate buffered saline (PBS, group B), low dose candidate vaccine (group C), medium dose candidate vaccine (group D), high dose candidate vaccine (group E).

Analysis of IgA antibody levels against RBD in saliva and serum of hamsters immunized with different doses of the candidate vaccine was performed on days 0, 14, 28, 35, 60, 120, 150, 180, and 210 after the first (IM), second (IM), and third dose (IN) of this candidate vaccine. The levels of IgA antibody in saliva did not show a statistically significant difference in different groups on day 35 (P>0.05). The results of IgA analysis in saliva showed a significant increase in IgA antibody level from day 14 after the third dose of the candidate vaccine (IN) in hamsters (P<0.001). Furthermore, 75% of animals showed a significant elevation of their saliva IgA antibody level in the vaccinated groups receiving the low, middle, and high doses on days 60 and 120 (P<0.05). There was no statistically significant difference between vaccine groups at 60, 120, and 210 days (P> 0.05) (**Figure 5**). Statistical analysis of serum levels of IgA antibody against RBD in hamsters immunized with different doses of candidate vaccine did not show a significant change at 14 and 28 days after first immunization compared to PBS and adjuvant groups (P>0.05), although a slight increase was observed. The increased IgA antibody of serum against RBD was significantly detectable one week after the second dose of the candidate vaccine (day 35) (P<0.05) and remained significantly higher in 80%-75% of animals receiving different doses of candidate vaccine until day 210 (P<0.001). From day 120 to 210, a non-significant decreasing trend was observed in all animals receiving the candidate vaccine, but it was still significantly higher than control groups (P<0.05) (**Figure 5**).

### RAZI-COV PARS Candidate Vaccine Induced Neutralizing Antibodies Against RBD Antigen

**Figure 6:**
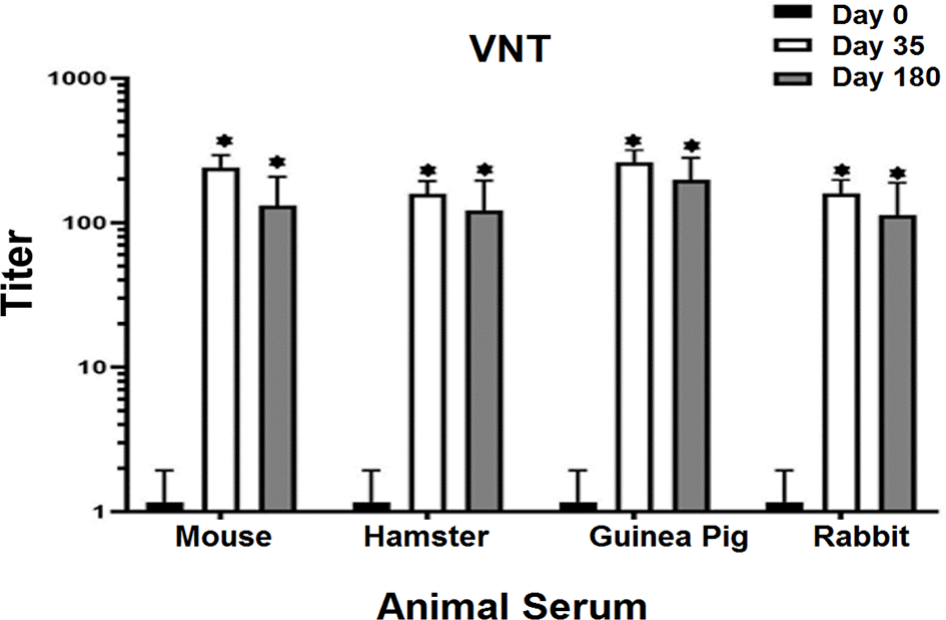
Virus Neutralizing Test (VNT) in vaccinated animals with middle dose of candidate vaccine. VNT has been done in Vero cell lines. All the vaccinated animals with middle dose of candidate vaccine were able to activate neutralizing antibodies after 14 days of the first vaccine booster. Excellent neutralizing titers of mice, hamsters, guinea pigs, and rabbits were observed in sera from the immunized animals on day 35 and 180 that showed significant difference with zero day. The seroconversion assay showed neutralizing antibodies on days 35 and 180 in 80 to 90% of the vaccinated animals. The control and placebo groups showed no ability to neutralize the virus. p values lower that 0.05 were considered to be statistically significant (*p<0.05). Data are presented as mean± SD. The levels of statistical significance for differences between test groups were determined using one-way ANOVA followed by Tukey’s *post hoc* test. Statistical comparison was done by comparing the vaccinated group on days 0 with days of 35 and 180.

The results of neutralization antibody assay showed that middle and high dose of candidate vaccine were able to neutralize virus in mice, hamsters, guinea pig, and rabbits on days 35 and 180 in 80% to 90% of animals. Serum samples in the control and placebo groups showed no ability to neutralize the virus. Excellent neutralizing titers of mice, hamsters, guinea pigs, and rabbits were observed in sera from the immunized animals on day 35 and 180 that showed extremely significant difference with zero day (P<0.001) (**Figure 6**). Our results clearly show that the current recombinant proteins could represent promising candidates for the development of a COVID-19 vaccine and can elicit very strong neutralizing activities against SARS-CoV-2 in a short period of time.

### Human ACE2 Protein (hACE-2) Binds Assay to RAZI-COV PARS Candidate Vaccine in Mice

There was a statistically significant difference in inhibiting ACE2 adhesion in the sera of vaccinated mice on days 35 with different doses of candidate vaccines in comparison with placebo and control groups (P<0.001). The lowest inhibitory effect was observed at high doses of candidate vaccine on day 35 (P<0.05). The placebo and control group showed no ability to inhibit ACE2 adhesion (**Figure 4S**).

### RAZI-COV PARS Candidate Vaccine Elicits RBD-Specific IgG2a/IgG1 Antibody Responses in Mice

The level of IgG2a and IgG1 antibody isotypes in murine sera were assessed to determine whether a dominant Thl or Th2 lymphocyte response is developed following immunization in mice. The results showed that low, middle, and high doses of RAZI-COV PARS vaccine candidate could stimulate either, or both, the IgG1 and IgG2a isotypes. Administration of middle and high doses of candidate vaccine resulted in a higher IgG2a/IgG1 ratio on day 35, indicating a Th1-dominant response in immunized mice (**Figure 5S**) (P<0.01).

### RAZI-COV PARS candidate vaccine elicits Th1 cytokines

For all animals euthanized on day 14 and day 35, the cellular immune response was characterized through the isolated splenocytes of mice immunized with RAZI-COV PARS candidate vaccine. In a cytokine release assay, TNFα levels showed a significant increase on days 14 and 35 following the immunization with different dose of candidate vaccine compared to the control and placebo group (P<0.05). Interleukin 2 (IL-2) levels showed a dose depending increase on day 35 after immunization with different dose of candidate vaccine, compared to the control and placebo groups. The level of interleukin 4 (IL-4) was not detectable on day 35 after the immunization of different groups with a low dose of RAZI-COV PARS candidate vaccine as well as in the control and placebo groups. A highly specific detection of IL-4 was possible in the middle and high doses groups of RAZI-COV PARS candidate vaccine. The level of Interleukin-17 (IL-17) was also detected in groups receiving low, middle, and high doses of RAZI-COV PARS candidate vaccine. As with the IL4, the highest amount was seen on day 35 following the injection of high doses of the RAZI-COV PARS candidate vaccine (P<0.05). The amount of the interleukin 6 (IL-6) in mice immunized with low, middle, and high doses of RAZI-COV PARS candidate vaccine was not detectable on days 14 and 35 after immunization and did not show significant differences with the control and placebo groups (P>0.05). Furthermore, the production of interferon-gamma (IFN-γ) showed a significant increase on days 14 and 35 after immunization of all groups receiving different doses of RAZI-COV PARS candidate vaccine when compared to the control and placebo groups (**Figure 6S**).

### RAZI-COV PARS Vaccine Candidate Increases Lymphocyte Proliferation

**Figure 7:**
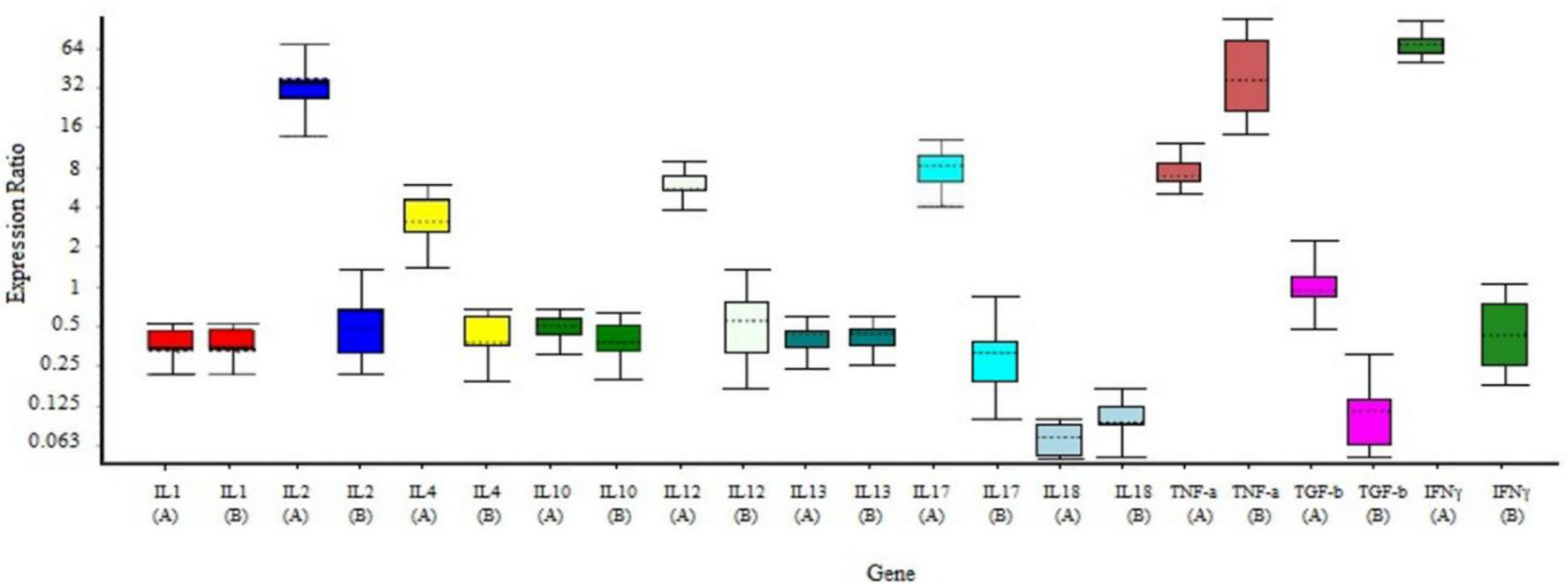
Gene expression profile of the splenocytes from immunized and control mice. Mice were primed-boosted with the middle dose of the RAZI-COV PARS vaccine (A, vaccine) or with the adjuvant RAS-01 only (B, placebo). On 35 days after immunization, mice were euthanized and splenocytes isolated and incubated with S protein for 24 hrs and total RNA was extracted. Gene expression was determined according to the ΔΔCt using IQ5 software. The results showed that the markers of the inflammation such as IL1 and IL18 had no changed in vaccinated group (P>0.05), moreover, the markers of Th1 responses such as IL-2, IL-12, IL-17, and IFNγ are significantly stimulated following vaccination (P<0.05), while the markers of Th2 responses such as IL-10, IL-4 and IL-13 were induced, but to a limited extent (P>0.05). Mean± SD is depicted in the bar graphs. The statistical significance was assessed using the Kruskal-Wallis test followed by the two-tailed Mann-Whitney test between the two groups; p values less than 0.05 were considered to be statistically significant.

**Figure 8:**
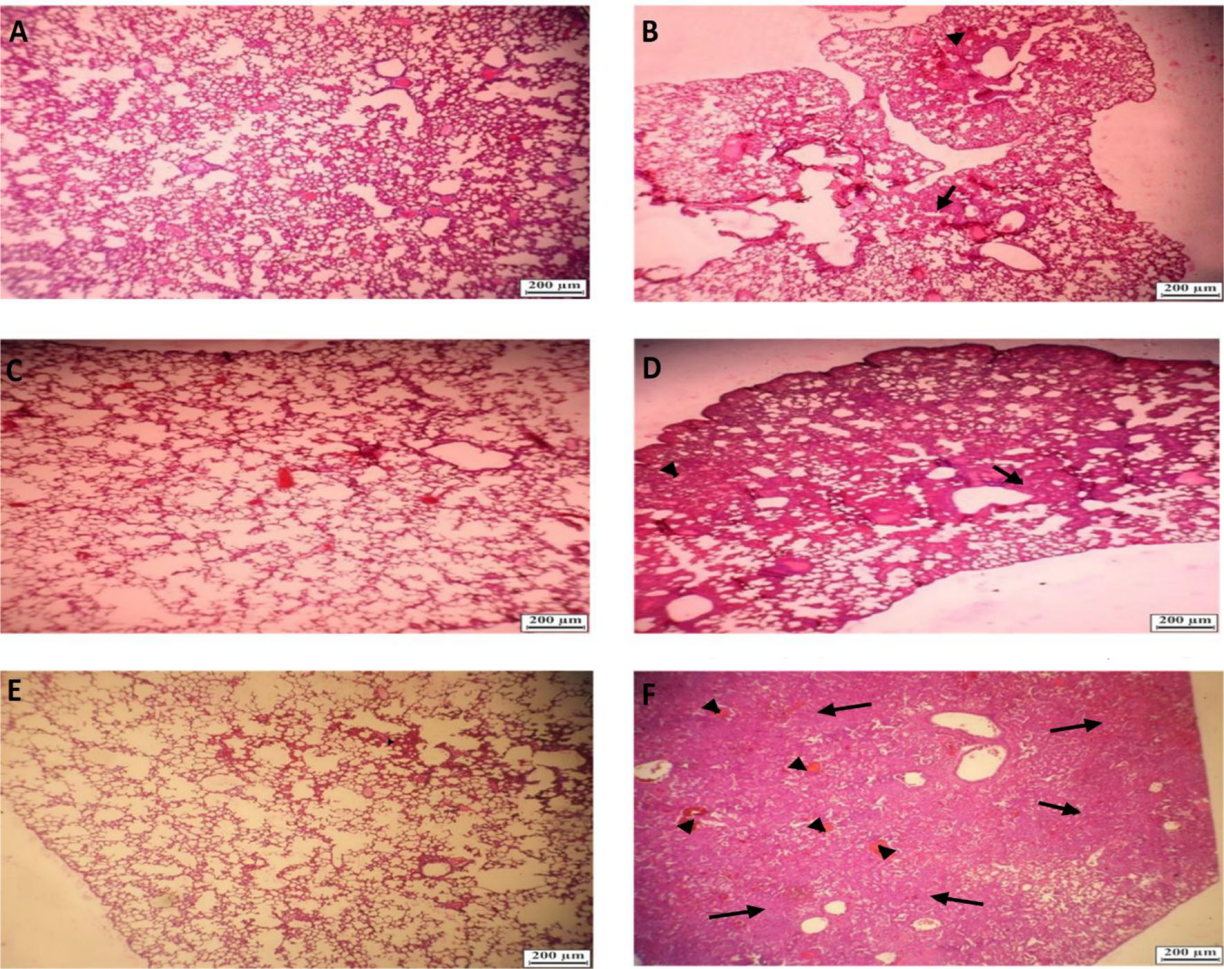
Histopathological examinations in lungs f of hamster from challenged animals on 3, 5, and 7 DPI (day post-infection). **(A)** Lungs of hamster from vaccinated group on 3 DPI showing alveolar septa with mild thickened (Hematoxylin and eosin × 40). **(B)** Lungs of hamster from non-vaccinated hamsters’ group on 3 DPI showing mild interstitial pneumonia (arrow) and small foci of hyperemia (arrowhead) (Hematoxylin and eosin×40). **(C)** Lungs of hamster from vaccinated hamsters challenged on 5 DPI showing foci of hyperemia (arrow) (Hematoxylin and eosin×40). **(D)** Lungs of hamster from non-vaccinated hamsters on 5 DPI showing moderate to severe interstitial pneumonia and lymphocytic infiltration (Hematoxylin and eosin× 40). **(E)** Lungs of hamster from vaccinated hamsters challenged on 7 DPI) Hematoxylin and eosin×40). **(F)** Lungs of hamster from non-vaccinated hamsters challenged on 7DPI showing severe interstitial pneumonia (arrow) and foci of hyperemia (arrowhead) (Hematoxylin and eosin×40).

**Figure 9:**
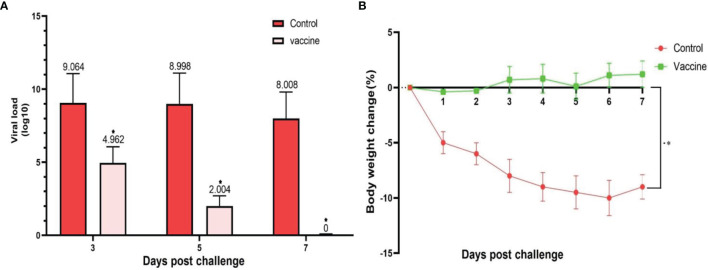
SARS-CoV-2 challenge of immunized hamsters (103 ± 20g) with middle (M) dose of candidate vaccine. **(A)** The viral load was calculated as TCID50%/mL. Lungs samples of the control group showed an average titer of 1.16×10^9^ TCID50/ml, 7.83×10^8^ TCID50/ml, and 10.2×10^7^ TCID50/ml on 3, 5 and 7 DPI, respectively. In the vaccinated groups with the middle dose of candidate vaccines, the lung viral titer was remarkably low compared to the control group as 9.17×10^4^ TCID50/ml, 10.2×10^3^, and 0 on 3, 5, and 7 DPI, respectively. However, the vaccinated group did not show any live virus titer in any of the specimens on 5 and 7 DPI. **(B)** The weight change between day 0 and day 7 post SARS-CoV-2 challenge of the vaccinated and control hamsters. There was a significant difference in weight between the RAZI-COV PARS vaccine and the control group during the period of observation on days 1 and 2 (P <0.05) as well as statistically very significantly on days 3 to 7 (P <0.01). Results are presented as the mean ± SD. Comparisons between control and vaccine group were made between the two groups by T-test (P < 0.05). *p* values lower that 0.05 were considered to be statistically significant (*p <0.05).

Percentage of lymphocyte proliferation in cell suspension after stimulation with RAZI-COV PARS candidate vaccine on day 35 showed a significant and dose-dependent increase after the immunization with different doses of candidate vaccine in comparison with the control and placebo groups (P<0.05). Furthermore, for all mice samples, we used Flow Cytometry to quantify intracellular cytokine IFNγ in CD3+/CD8+, CD3+/CD4+ and also the percentage of CD3+/CD62L+, CD3+/CD27+ and CD3+/CD127+ cells after immunization. On day 35, high percentages of CD3+/CD8+ and to a slightly lesser extent CD3+/CD4+ cells were detected (**Figures 7S, 8S, 9S**). However, a decrease in CD62L and an increase in CD27 and CD127 of the mice immunized with RAZI-COV PARS candidate vaccine was seen compared with the control groups.

### RAZI-COV PARS Candidate Vaccine Increases Cellular Immune Responses

As depicted by our above-presented immunogenicity studies, the optimal dose for induction of the specific immune responses in mice is middle doses of RAZI-COV PARS candidate vaccine. Here, we used the group receiving this optimal dose to evaluate the cellular immune response through real-time PCR (**Table 2**). Our results showed that the markers of the inflammation such as IL1 and IL18 had no change in vaccinated group (P>0.05), moreover, the markers of Th1 responses such as IL-2, IL-12, and IFNγ are significantly stimulated following vaccination (P<0.05), while the markers of Th2 responses such as IL-10, IL-4, and IL-13 were induced, but to a limited extent (P>0.05) (**Figure 7**).

**Table 2:** Primers used in this study for evaluating the genes expression including GAPDH, β-actin, IL-1, IL-2, IL-4, IL-10, IL-12p40, IL-13, IL-17, IL-18, TNF-α, INF-γ, and TGF through an iCycler from mouse splenocytes.

Genes	Primer Forward	Primer Reverse
**GAPDH**	5’-TGA GCA AGA GAG GCC CTA TC -3’	5’-AGG CCC CTC CTG TTA TTA TG -3’
**B-actin**	5’-AGA GCG GGC CTT GAG AAA AG -3’	5’-TGG AGA GCC TGG ATT GTC ATC -3’
**IL-1**	5’-CAA CCA ACA AGT GAT ATT CTC CAT G -3’	5’-GAT CCA CAC TCT CCA GCT GCA -3’
**IL-2**	5’-CCT GAG CAG GAT GGA GAA TTA CA -3’	5’-TCC AGA ACA TGC CGC AGA G -3’
**IL-4**	5’-ACA GGA GAA GGG ACG CCA T -3’	5’-GAA GCC CTA CAG ACG AGC TCA-3’
**IL-10**	5’-GGT TGC CAA GCC TTA TCG GA -3’	5’- ACC TGC TCC ACT GCC TTG CT -3’
**IL-12p40**	5’-GGA AGC ACG GCA GCA GAA TA -3’	5’- AAC TTG AGG GAG AAG TAG GAA TGG -3’
**IL-13**	5’-GGA GCT GAG CAA CAT CAC ACA -3’	5’-GGT CCT GTA GAT GGC ATT GCA -3’
**IL-17**	5’-GCT CCA GAA GGC CCT CAG A -3’	5’-AGC TTT CCC TCC GCA TTG A -3’
**IL-18**	5’-CAG GCC TGA CAT CTT CTG CAA -3’	5’-TCT GAC ATG GCA GCC ATT GT -3’
**TNF-α**	5’-CAT CTT CTC AAA ATT CGA GTG ACA A -3’	5’-TGG GAG TAG ACA AGG TAC AAC CC -3’
**INF-γ**	5’-TCA AGT GGC ATA GAT GTG GAA GAA -3’	5’-TGG CTC TGC AGG ATT TTC ATG -3’
**TGF**	5’-TGA CGT CAC TGG AGT TGT ACG G -3’	5’-GGT TCA TGT CAT GGA TGG TGC -3’

### Vaccine Efficacy and Pathological Findings in Lungs After Virus Challenge

The lungs of the vaccinated groups appeared normal on 3, 5, and 7 DPI (day post-infection) (**Figures 8A, C, F**) grossly, whereas, on 5 DPI, the lungs of the non-vaccinated group showed moderate to severe interstitial pneumonia and lymphocytic infiltration and small foci of hyperemia (**Figures 8B, D**). On histopathological examination, lung sections from non-vaccinated animals group showed inflammatory infiltration including macrophages, lymphocytes, and hemorrhage on 3, 5, and 7 DPI. Some necrotic areas were also seen in the parenchyma. Perivascular infiltration of inflammatory cells was shown in blood vessels. Inter alveolar spaces were proliferative (**Figures 8B, D**). Vaccinated animals did not show any histopathological evidence of pneumonia except a few mild thicknesses of alveolar septa on 3 DPI (**Figure 8A**), foci of hyperemia on 5 DPI (**Figure 8C**), and the foci of hyperemia, and mildly thickened of alveolar septa at 7 DPI (**Figure 8E**). Lungs samples of the control group showed an average titer of 1.16×10^9^ TCID50/ml, 7.83×10^8^ TCID50/ml, and 10.2×10^7^ TCID50/ml on 3, 5, and 7 DPI, respectively. In the vaccinated groups with the middle dose of candidate vaccines, the lung viral titer was remarkably low compared to the control group as 9.17 × 10^4^ TCID50/ml, 10.2×10^3^, and 0 on 3, 5, and 7 DPI, respectively. However, the vaccinated animals did not show any live virus titer in any of the specimens on 5 and 7 DPI (**Figure 9A**). Weights of Syrian hamsters were recorded daily for 7 days after the virus challenge to assess vaccine efficacy. Only animals immunized with the RAZI-COV PARS vaccine showed a slight increase in body weight during 7 days of observation, with control groups losing weight. Our results also showed that COVID-19 negatively impacts body weight in the control group (**Figure 9B**). Furthermore, there was a decrease in body weight of the control group reaching 10% reduction on day 6 and 9.8% reduction on Day 7. There was a significant difference in weight between the RAZI-COV PARS vaccine and the control group during the period of observation on days 1 and 2 (P <0.05) as well as statistically very significantly on days 3 to 7 (P <0.01).

## Discussion

In the present study, the preliminary assessment of the safety and protection of the optimal dose of the RAZI-COV PARS candidate vaccine was performed in different animal models. The cellular and humoral immune responses of BALB/c mice, Syrian hamsters, guinea pigs, and NZW rabbits following the administration of different doses of the RAZI-COV PARS candidate vaccine were carefully examined. The protective immunity of a vaccine candidate based on the S-Trimer of SARS-COV-2 has recently been shown in rats, rabbits, and mice as well as in nonhuman primates (27). Similar to our study, the safety profile of BBV152A, BBV152B, and BBV152C candidate vaccines was evaluated in different animal models including rats, rabbits, and mice (40). In the current study, the mortality, general clinical observations, body weight and food intake, clinical indicators, hematology examination, blood chemistry, and pathological examination of vital organs showed that this vaccine meets scientific standards for safety, quality, and efficacy. The change in the body weight is a very sensitive indicator of toxicity as referred in ICH guidelines. Throughout the study, hematology examination in either fasting or non-fasting conditions at variable time points were evaluated by Hct, Hb, erythrocyte count, total and differential leukocyte counts, MCH, and MCV and showed no remarkable significant differences in several hematological indicators regarding the control group (P > 0.05). The evaluation of CT, PT, PTT, and PLT in animal models also declines clotting concerns. Furthermore, the weight changes for vital organs were reported to be statistically non-significant in all exanimated groups, particularly for the brain weight ratios as a more reliable indicators of organ-directed toxicity. Similar to our study, a different study showed integrated analysis of clinical signs, animal and organ weight, food and water intake, behavior, hematology, and blood chemistry allows assessing toxicity associated with repeated exposure to vaccine (8, 41, 42). Several protein-based vaccines have promising results exhibiting great safety profiles and high levels protection through the T cell responses and virus neutralizing antibodies both in nonhuman primates and in early studies in humans (8, 43–45). Our study showed that RAZI-COV PARS candidate vaccine induced a strong specific IgG antibody response against S1, RBD, and NTD antigens as well as both salivary and serum RBD-specific IgA antibodies, thereby leading to a robust antibody-mediated neutralizing activity. RAZI-COV PARS candidate vaccine is different from the previous protein-based vaccines of COVID-19, because it contains three immunogenic components of two subunits, S1 and S2 and trimeric S protein of SARS-CoV-2 together. S1 contains an exposed RBD that binds ACE2 receptors while S2 is necessary for fusion of host membranes and virus, which is not fully exposed until after receptor binding (46). The RBD is a poorly conserved region of S and represents a promising antigen for induction CoV-specific antibodies, while S2 is highly more conserved across coronaviruses that induce more cross-reactivity CoV-specific antibodies compared to S1 (47, 48), revealing beneficial for vaccine design (49). A specific fragment in the S2 subunit of SARS-CoV-2 [corresponding to residues fusion peptide (FP) (residues 795-848) and HR2 (residues 1127-1177)] was also found to stimulate neutralizing antibodies against SARS-CoV-2 that elicit a longer-lasting and stronger memory response, and decrease the likelihood of sequence-altering mutations that decrease the efficacy of vaccine (48, 50). Furthermore, trimeric S protein with two different adjuvants induced high levels of Th1-biased cellular immune responses and neutralizing antibodies in animal and human models and induce suitable protection from SARS-CoV-2 challenge (27, 41). Therefore, we use a large panel of immunogenic components of two subunits, S1 and S2 and trimeric S protein of SARS-CoV-2 as antigen for reliable induction of SARS-CoV-2–specific antibodies. As indicated by previous recombinant based COVID-19 vaccine such as Clover Biopharmaceuticals, S-Trimer demonstrated strong neutralization against the different SARS-CoV-2 variants and strain, specifically the UK variant (B.1.1.7), Brazil variant (P.1), and South African variant (B.1.351) (51). The antibody-binding activity and neutralizing capacity were substantially similar to previously reported studies in rodents vaccinated with a whole-inactivated virus (52), recombinant proteins (51, 53), DNA (54), or adenovirus vector vaccines (55), all reporting a protective humoral response in the vaccinated groups. Moreover, high levels of S1, RBD, and NTD specific-antibodies were detected within three weeks after the low, middle, and high doses of candidate vaccines, revealing that the production of specific IgG and IgA antibodies is significantly stimulated after the first injection of RAZI-COV PARS candidate vaccine. S1, RBD, and NTD-specific antibodies have been detected as early as 28 days after the first dose of vaccination and showed a remarkable increase following the second dose of vaccination from day 35 onwards. The ability of the secreting S1 and S2 proteins to elicit SARS-CoV specific IgG and IgA antibodies is also dependent on the suitable expression of antigens with accurate conformation and post-translational modification such as oligomerization and glycosylation, all influencing the complex folding which occurs during protein sorting and secretion (56). The vaccination with the RAZI-COV PARS was able to stimulate the production of neutralizing antibodies in BALB/c mice, Syrian hamsters, guinea pigs, and NZW rabbits on days 35 and 180 in 80 to 90% of animals receiving two doses of the RAZI-COV PARS candidate vaccine. Furthermore, a boost vaccination with this vaccine resulted in a significant increase in SARS-CoV-2 virus-neutralizing antibody titers. No specific neutralizing antibodies against SARS-CoV-2 were shown in control and placebo groups. In humans, the correlation between the production of neutralizing antibodies and the protection against the SARS-CoV-2 infection is now well established (57, 58), although there is still evidence regarding the long-term protection against reinfection with SARS-CoV-2 (59). For the evaluation of neutralizing antibodies, the ability of antibodies to block SARS-CoV2 infection is examined (60). Different vaccines such as BBIBP-CorV (Sinopharm, Beijing CNBG) vaccine (61), Rabies virus-based COVID-19 vaccine CORAVAX™ (62), and Sputnik V vaccine (63) also led to the production of significant amounts of neutralizing antibodies which appeared to correlate with an elevated protection and decreased susceptibility to the COVID-19 disease. Comparison of neutralizing antibodies elicited by SARS-CoV-2 adenoviral vector and mRNA vaccine exhibited that Ad26.COV2.S-expressed antibodies revealed low neutralizing capacity in a significant portion of vaccinated individuals whereas mRNA-1273 and BNT162b2-elicited antibodies displayed a modest neutralization against different variants of SARS-CoV-2 including Lambda, Delta, Delta plus, and Beta (64). However, RAZI-COV PARS candidate vaccine induced a significant increase of virus-neutralizing antibody titers. A limitation of our study is that the cross-neutralizing ability of the specific antibodies induced by the RAZI-COV PARS candidate vaccine still needs to be investigated as the neutralization experiments that have been exclusively performed using an isolated wild type of SARS-CoV-2. The analysis of IgA antibody levels against RBD in saliva and sera showed a significant increase in IgA antibody levels from day 14 after the third dose of the candidate vaccine (IN) in hamsters. Furthermore, 75% of the animals in the vaccinated groups showed a significant elevation of their IgA antibody titers after receiving the middle and high doses of candidate vaccine on days 60 and 120. The group receiving a low dose of candidate vaccine experienced a non-significant decreasing trend of their IgA antibody levels against RBD in saliva from day 60, but these levels appeared to be still significantly higher than those observed on day 0 and the control groups. The most important issue that has been neglected in previous vaccines is the lack of stimulation of the immune system against the virus in the upper respiratory tract (URT). RAZI-COV PARS candidate vaccine with the intranasal spray (third dose) was able to induce high levels of IgA antibody that largely compensate for this defect. We were also able to detect IgA antibodies in URT which are considered a major barrier against viral infection by impeding viral shedding, limiting the degree of spread and level of pathology (65). It is important to note that IgA antibodies are associated with the expansion of the IgA plasmablasts with mucosal homing characteristics and manage the early response against specific antigens of SARS-CoV-2 in the serum, saliva, and bronchoalveolar lavage fluids. IgA concentrations in the saliva and serum peaked after the administration of the third dose of the intranasal vaccine but persisted for several more months in the saliva and serum. It has been reported that IgA concentrations in saliva were more potent in comparison with IgG to neutralize SARS-CoV-2 (66) and were effective in eliminating invading SARS-COV-2 during the early stages of SARS-CoV-2 infections (67). We also identified the IgG subclasses induced by RAZI-COV PARS candidate vaccine and a higher IgG2a/IgG1 ratio was noted after the second dose of vaccine on day 35 with predominant response of IgG2a, indicating a Th1-dominant response in immunized mice. Other studies using a mice model also found a higher IgG2a/IgG1 ratio in the group receiving the inactivated SARS-CoV-2 vaccine-BBV152 (40). Our results also found the secretion of different pro-inflammatory cytokines such as IL-17 and TNF-α in the splenocytes from immunized mice after the first and second dose of RAZI-COV PARS candidate vaccine, suggesting a hyperinflammatory response by recruiting T and B cells as well as macrophages in the serum. Moreover, IL-2 and IFN-γ as the main biomarkers of SARS-CoV-2 specific cellular response showed a dose-dependent increase after the first and second dose, respectively. Similarly, mRNA vaccine of BNT162b2 elicited a cytokine signature featuring IFN-γ, and CXCL10 (68), which were followed by a significant increase in TNF-α after the second vaccination. Our results also highlighted a limited release of IL-4 in the mice treated with the middle dose of RAZI-COV PARS candidate vaccine, suggesting the low activation of Th2 immune response. IL-2 is a growth factor for production of memory T cells and the cellular expansion of specific T cells (69). Intracellular staining reported that both CD4+T and CD8+T cells are the origins of this cytokine but the main generation of this cytokine carried out through CD4+T cells (70, 71). Importantly, it has been revealed that SARS-CoV-2 specific antibodies correlates with IFN-γ and IL-2 regulated cellular response (69). Our results also confirm a release of IL-17 in the mice groups under low, middle, and high dose of RAZI-COV PARS candidate vaccine, revealing a specific subset of T helper cells that selectively produce the cytokine Th17 (72). Moreover, IL-17 can be generated by different immune cells such as natural killer (NK) cells, γβT and αβ -cells. The expression of receptors for IL-17a and IL-17F take place on mucosal epithelial cells (73). The cytokine IL-17 is critical for the maintenance of mucosal homeostasis. Therefore, our results confirmed that the release of IL-17 in the mice immunized with the RAZI-COV PARS candidate vaccine is very important to the epithelial integrity of the host and promotes antimicrobial activity. Moreover, using flow cytometry, the number of the activated memory lymphocytes such as CD3/CD8, CD3/CD4, CD3/CD27, and CD3/CD127 was found to be increased in the mice treated on day 35, revealing that the RAZI-COV PARS candidate vaccine was able to stimulate the efficient dendritic cell cross-delivery of spike protein to CD8 T cells (74). However, a decrease in CD62L and an increase in CD27 and CD127 in the vaccine groups versus the control groups indicate a change in naive T cells to specific effector and memory T cells. In a study that revealed the expression of CD127, the alpha chain of the IL-7 receptor could be applied to reveal the memory precursor effector cells (MPECs) that display increased ability to form short-lived effector cells (SLECs) and long-lived memory cells (75). Another investigation also reported that expression of CD27 could identify effector cells that were more likely to survive contraction (76). Importantly, the increase of CD8 T cells was related to remarkable *in vivo* activity of cytotoxic T lymphocyte against spike targets, indicating that RAZI-COV PARS candidate vaccine should be able to powerfully control SARS-CoV-2 infection along with induction of cytotoxic T lymphocyte that was able to effectively recognize and kill any residual cells of virus in the body as well as induction of neutralizing antibody. The surface markers of lymphocytes comprised of CD8 (T cytotoxic cells), CD4 (T helper cells), and CD3 (Pan T-cell marker) (77). Significant CD4 down-regulation was found in high portion of CD3/CD4, which was negatively connected to intracellular cytokine secretion. Similarly, immunization with Advax-SM adjuvanted rSp stimulated a high level of memory T cells of CD4+ and CD8+, which were not reported in mice treated with recombinant spike protein alone (78). From T cell activation markers, we also showed a high level of CD4+ T cells up-regulating of IFN-γ and IL-4, likely revealing high percentage activity of T follicular helper cells, which also showed a supportive role in the production of antibody-producing plasma cells and long-lived memory B cells (79). Our results also confirmed that the gene expression of Th1 biomarkers, including IL-2, IL-12, IL-17, and IFNγ in mice immunized with RAZI-COV PARS candidate vaccine are largely stimulated while those associated with Th2 responses such as IL-4 has a limited induction. Similarly to our results, another study also confirmed that mice vaccinated with the Advax-SM adjuvanted rSp induced a strong Th1 response characterized by increased TNF-a, IFN-c, and IL-2 secreting anti-spike T cells, a switch from IgG1 to IgG2 and IgG3 isotypes, and CTL killing of spike-labelled target cells (78). However, immunization of mice with recombinant spike protein formulated with alum adjuvant (or alone) stimulated mostly T cell response and Th2 antibody against spike protein, with no neutralizing induction against the variant of B.1.1.7 virus and lower contents of neutralizing antibody responses against the wild type virus along with no *in vivo* activity of CTL against spike-labelled targets (78). *In vitro* binding assay of hACE-2 in our study revealed that a statistically significant difference in inhibiting hACE-2 adhesion in the sera of vaccinated mice on days 35 and 65 under different doses of candidate vaccines. According to these results, the lowest inhibitory power was observed at high doses of candidate vaccine on day 35, which continued until day 65. The adjuvant group showed no ability to inhibit hACE-2 adhesion, thereby indicating that the inhibitory power of hACE-2 adhesion was associated with the administration of the recombinant proteins. With the use of this humanized ACE-2 antigen and the inhibition created by antibodies after immunization with RAZI-COV PARS candidate vaccine, it can be expected that this inhibition will be fully realized in human tissues.

## Conclusion

The results of our study indicated that the RAZI-COV PARS candidate vaccine induced the high humoral and cellular immune responses in different animal models. The best results were achieved using the middle dose of RAZI-COV PARS candidate vaccine. We also showed that the intranasal administration of the candidate vaccine can activate the immune system of the UTR and can prevent the shedding of the virus. Nasal vaccine of COVID-19 is feasible and could help block viral infections and slow transmission.

## Data Availability Statement

The original contributions presented in the study are included in the article/**Supplementary Material**. Further inquiries can be directed to the corresponding author.

## Ethics Statement

The animal study was reviewed and approved by Institutional Biosafety Committee and Institutional Animal Ethics Committee of the Razi Vaccine and Serum Research Institute (RVSRI), Karaj, Iran (IR.RVSRI.REC.1399.001).

## Author Contributions

SB designed the RAZI-COV PARS vaccine and RAS-01. SB, AE-h, MF, and MN conceived and designed the study. SB, AM, AR, MH, SR, MT, and MB performed vaccine production. SB, MN, and ME performed the planning of animal experiments. SB, MFM, ZSH, ML, AK, and ME performed the animal experimentation. SB, ZSH, MB, MT, SR, MM, and AE-h performed the laboratory work planning and data analysis. SB, MB, MT, SR, MM, and AK performed sample processing in the laboratory. SB, MD, MF, MN, and ME have drafted the manuscript. SB, AE-h, MFM, MS, MD, ZMH, ML, and FR substantively revised it. All authors reviewed the manuscript and agree to its contents. All authors contributed to the article and approved the submitted version.

## Funding

This study was supported by the grant 01-18-18-117-99035 from the Razi Vaccine and Serum Research Institute (RVSRI); Agricultural Research, Education and Extension Organization (AREEO), Karaj, Iran.

## Conflict of Interest

The authors declare that the research was conducted in the absence of any commercial or financial relationships that could be construed as a potential conflict of interest.

## Publisher’s Note

All claims expressed in this article are solely those of the authors and do not necessarily represent those of their affiliated organizations, or those of the publisher, the editors and the reviewers. Any product that may be evaluated in this article, or claim that may be made by its manufacturer, is not guaranteed or endorsed by the publisher.
