# Supplementary material for: Safety and Efficacy of Combined Intramuscular/Intranasal RAZI-COV PARS Vaccine Candidate Against SARS-CoV-2: A Preclinical Study in Several Animal Models
Source: Front Immunol. 2022 May 26;13:836745. doi: 10.3389/fimmu.2022.836745 (PMC9179012; doi:10.3389/fimmu.2022.836745)
Supplement: Supplementary file 1 [file DataSheet_1.docx]

**Supplementary Data:**

Journal name: [**Frontiers in Immunology**](https://loop.frontiersin.org/journal/276)

Manuscript Title: **Immunogenicity and efficacy of intramuscular-intranasal RAZI-COV PARS vaccine candidate against SARS-CoV-2: A Preclinical Study in animal models**

**The name(s) of the author(s):** **Seyed Reza Banihashemi*^1^, Ali Es-haghi^2^, Mohammad Hossein Fallah Mehrabadi^3^, Mojtaba Nofeli^4^, Ali Rezaei Mokarram ^5^, Alireza Ranjbar^6^, Mo Salman^7^, Monireh Haji Moradi^1^, Seyad Hossein Razaz^1^, Maryam Taghdiri ^1^, Mohsen Bagheri ^2^, Maryam Dadar ^4^,** **Zuhair Sarraf Hassan^10^, Mohammad Eslampanah ^8^, Zahra Salehi Najafabadi ^4^, Mohsen Lotfi ^9^, Akbar Khorasani ^4^, Fereidoon Rahmani ^2^**

**The affiliation(s) and address (es) of the author(s)**

1. Department of immunology, Razi Vaccine and Serum Research Institute, Agricultural Research, Education and Extension Organization (AREEO), karaj, Iran
2. Department of Physico Chemistry, Razi Vaccine and Serum Research Institute, Agricultural Research, Education and Extension Organization (AREEO), karaj, Iran
3. Department of epidemiology, Razi Vaccine and Serum Research Institute, Agricultural Research, Education and Extension Organization (AREEO), karaj, Iran
4. Department of research and development, Razi Vaccine and Serum Research Institute, Agricultural Research, Education and Extension Organization (AREEO), karaj, Iran
5. Department of QA, Razi Vaccine and Serum Research Institute, Agricultural Research, Education and Extension Organization (AREEO), karaj, Iran
6. Institute of Interventional Allergology and Immunology, Bonn / Cologne, Germany
7. Animal Population Health Institute of College of Veterinary Medicine and Biomedical Sciences, Colorado State University, Fort Collins, CO 80523-1644, USA
8. Department of Pathology, Razi Vaccine and Serum Research Institute, Agricultural Research, Education and Extension Organization (AREEO), karaj, Iran
9. Department of Quality Control, Razi Vaccine and Serum Research Institute, Agricultural Research, Education and Extension Organization (AREEO), Karaj, Iran
10. Department of Immunology, School of Medical Sciences, Tarbiat Modares University, Tehran, Iran, Tehran

**The e-mail address, telephone and fax numbers of the corresponding author**

***Corresponding authors: Seyed Reza Banihashemi**

Razi Vaccine and Serum Research Institute (RVSRI); Agricultural Research, Education and Extension Organization (AREEO), Karaj, Iran.

**[ORCID: 0000-0002-9025-915X](https://orcid.org/0000-0002-9025-915X)**


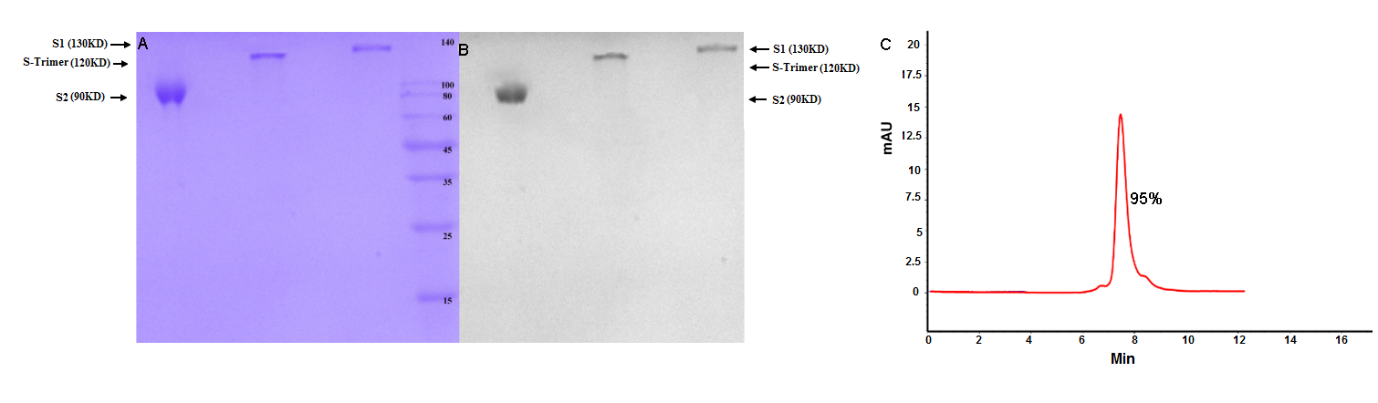


Figure1S. High-level expression and characterization of recombinant S1, S2 and S Trimer. A) Analysis of the purified recombinant antigens through the SDS-PAGE gel. B) Analysis of the purified recombinant antigens through the western blotting. C) HPLC analysis of purified recombinant S trimer on Bio SEC-3 (7.8 mm ID 30.0 cm L, 3 µm, 300 Å) column. SEC-HPLC performed as mobile phase; 150 mM sodium chloride, 50 mM phosphate buffer, adjusted to pH8, injection volume: 100 μl, flow-rate: 1 mL/min, and detection wavelength: 280 nm.


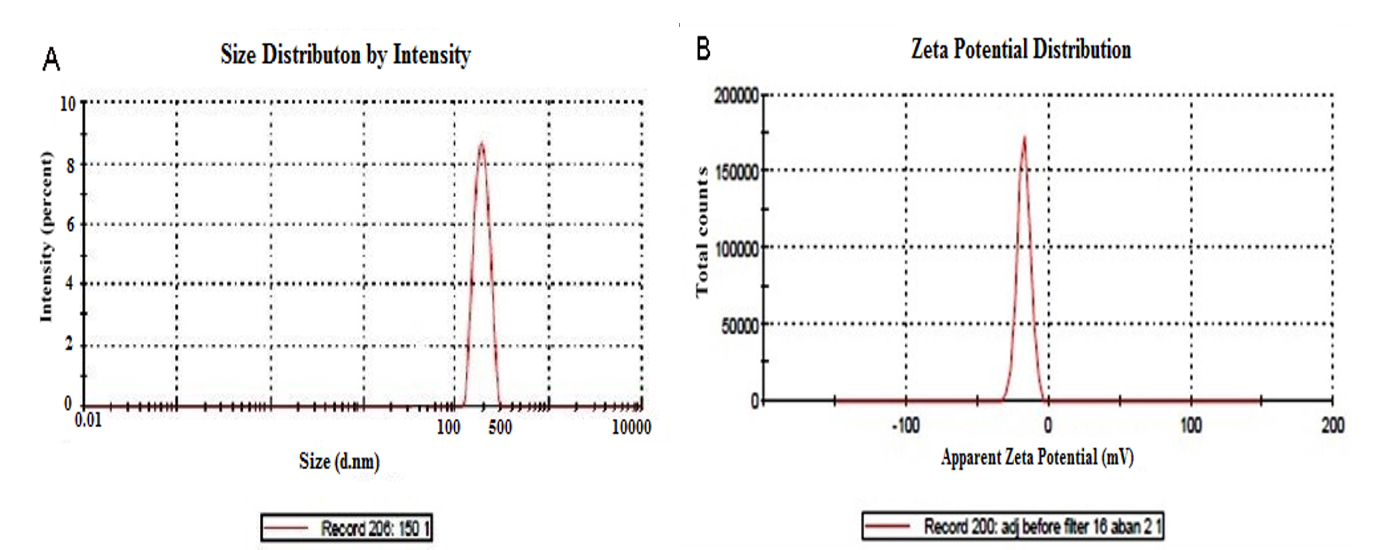


Figure2S. The size of the vaccine particle. A) The particle size is in the range of 200-500 nm through the DLS device and B) The zeta potential distribution represents that the particle charge of vaccine is negative.


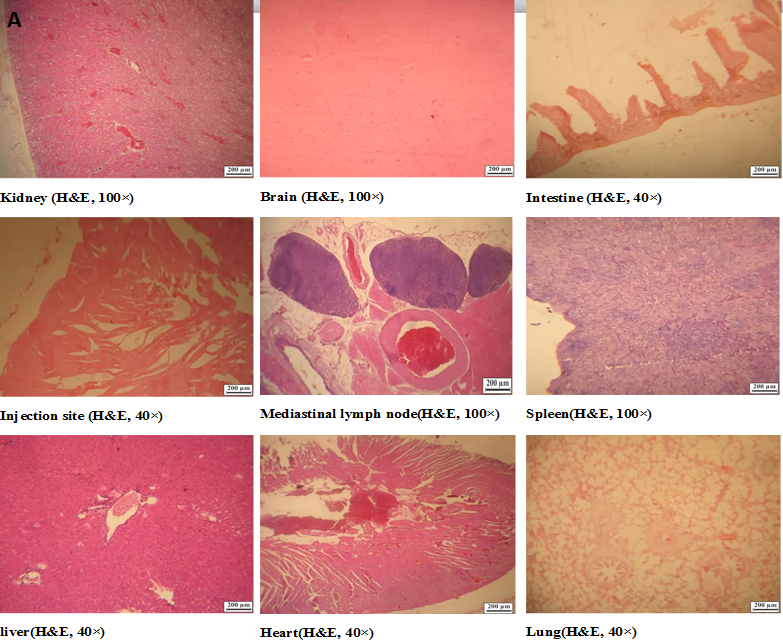


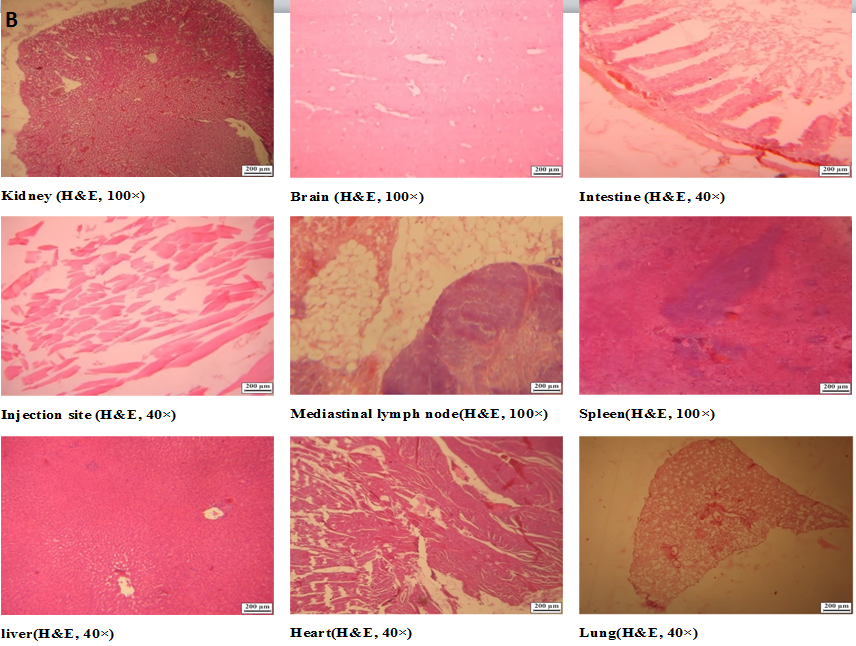


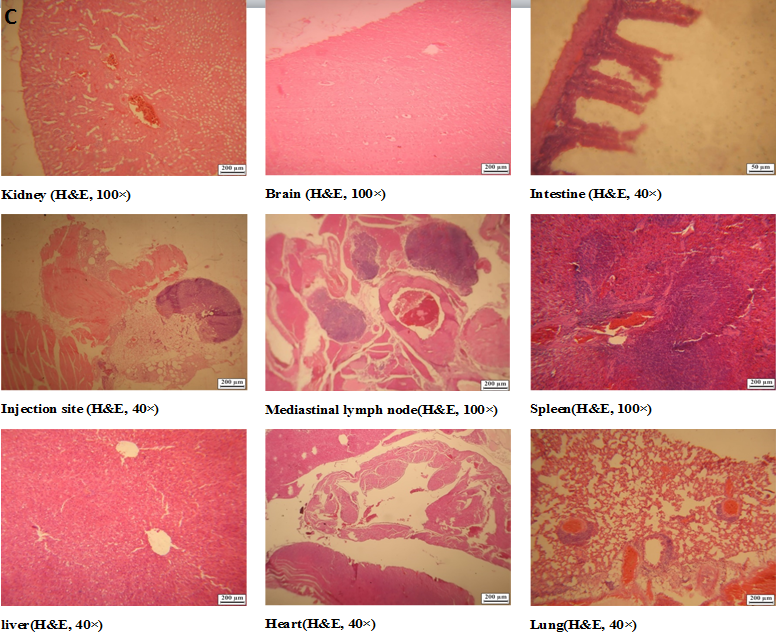


Figure 3S. Histological examinations in BALB/c mice tissues in [0.5µg (low), 1 µg (middle) and 2 µg(high)] dose of candidate vaccine. The results showed no significant lesions in the kidney, brain, intestine, injection site, mediastinal lymph node, spleen, liver, heart, and lung tissues of low, middle and high dose of candidate vaccine. A) Low dose of candidate vaccine. B) Middle dose of candidate vaccine. C) High dose of candidate vaccine.


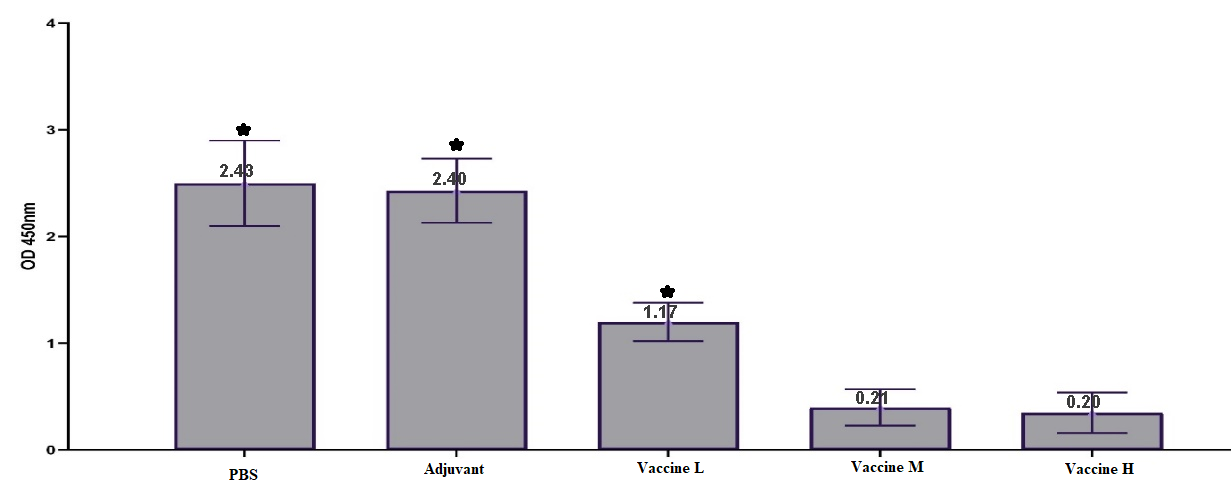
 Figure 4S: ACE-2 binds assay in mice immunized with candidate vaccine. The ACE-2 binding is significantly different in immunized groups with candidate vaccine. The lowest inhibitory effect was observed at high doses (Vaccine H) of candidate vaccine on day 35 (P<0.05). The adjuvant (placebo) and control (PBS) group showed no ability to inhibit ACE2 adhesion. Statistical comparison was done by comparing the vaccinated group with the the adjuvant (placebo) and control (PBS) groups as control. p values were calculated with Student’s *t* test. P values less than 0.05 were considered to be statistically significant (* p <0.05). The levels of statistical significance for differences between test groups were determined using one-way ANOVA followed by Tukey’s *post hoc* test. High dose of candidate vaccine=Vaccine H, Low dose of candidate vaccine=Vaccine L, Middle dose of candidate vaccine=Vaccine M. Asterisks denote significant differences between the adjuvant (placebo) and control (PBS) group and the vaccine groups (Vaccine L, Vaccine M and Vaccine H).


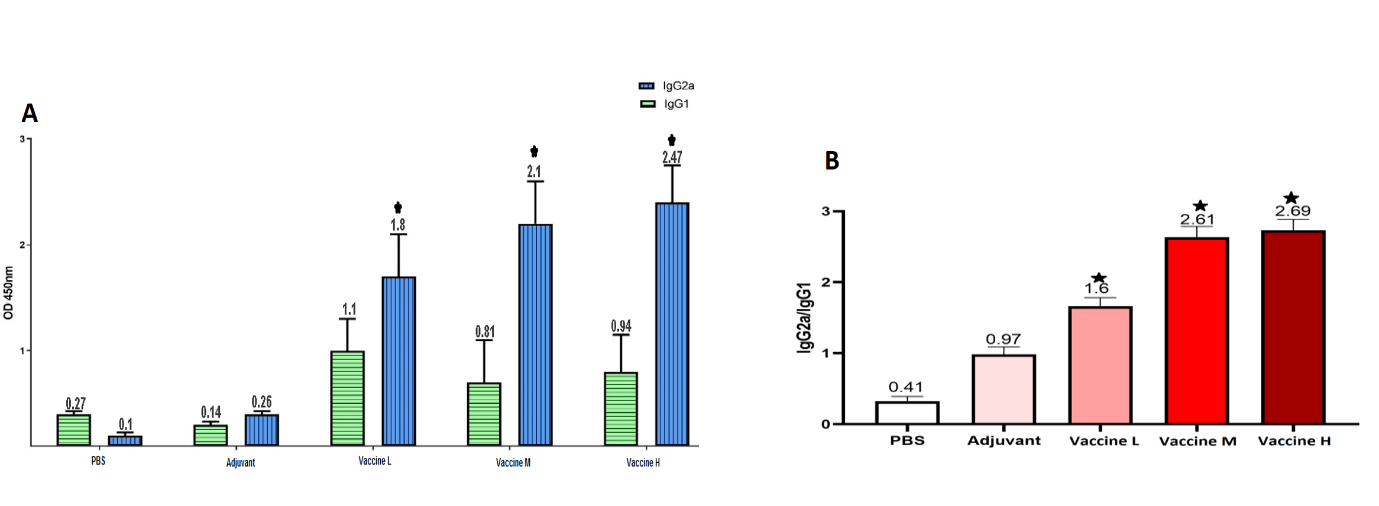


Figure 5S: IgG1 and IgG2a assay in mice immunized with candidate vaccine. A) The amount of specific IgG2a isotype in vaccinated groups that is completely higher than IgG1 isotype. This is a dose dependence increases based to antigen content. B) The ratio IgG2a/IgG1 in mice immunized with candidate vaccine. p values were calculated with Student’s *t* test. P values less than 0.05 were considered to be statistically significant (* p <0.05). The levels of statistical significance for differences between test groups were determined using one-way ANOVA followed by Tukey’s *post hoc* test. High dose of candidate vaccine=Vaccine H, Low dose of candidate vaccine=Vaccine L, Middle dose of candidate vaccine=Vaccine M. Asterisks denote significant differences between the adjuvant (placebo) and control (PBS) group and the vaccine groups (Vaccine L, Vaccine M and Vaccine H).


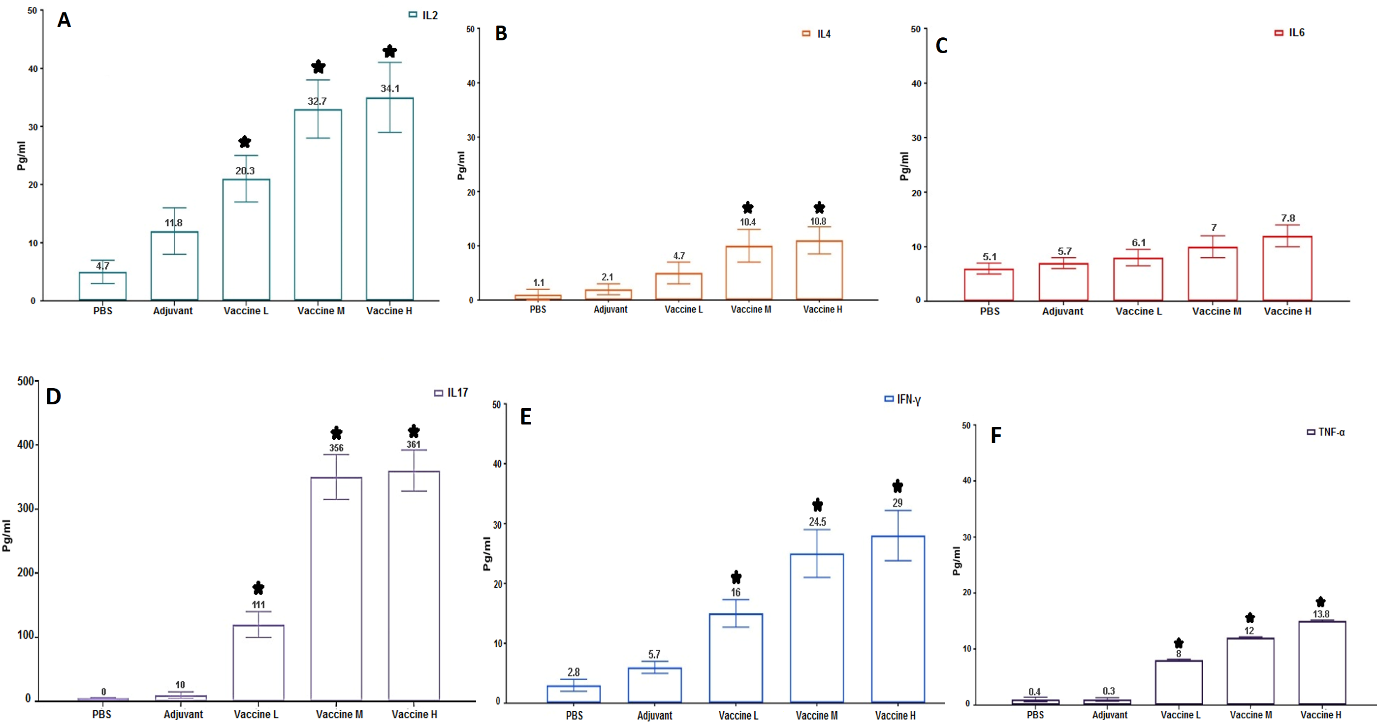


Figure 6S. Evaluation of cytokine level by ELISA. The increase of Th1 cytokines such as IL2, TNF, IFN-γ in comparison with IL4 and IL6 interleukins has been shown in vaccine groups. A) IL2 levels showed a dose depending increase following the immunization with high, middle and low candidate vaccine compared to the PBS (control) and adjuvant groups (placebo) (P<0.05). B) The level of IL4 showed a dose depending increase following the immunization with high and middle dose of candidate vaccine as well as in the PBS (control) and adjuvant groups (placebo) (P<0.05). C) IL6 in mice immunized with low, middle and high doses of candidate vaccine was not detectable significantly(P<0.05). D) IL17 was significantly detected in mice receiving low, middle and high doses of candidate vaccine(P<0.05). E) IFN-γ was significantly detected in mice receiving low, middle and high doses of candidate vaccine(P<0.05). F) TNFα levels showed a significant increase following the immunization with high, middle and low candidate vaccine compared to the PBS (control) and adjuvant groups (placebo) (P<0.05). p values were calculated with Student’s *t* test. P values less than 0.05 were considered to be statistically significant (* p <0.05). The levels of statistical significance for differences between test groups were determined using one-way ANOVA followed by Tukey’s *post hoc* test. High dose of candidate vaccine=Vaccine H, Low dose of candidate vaccine=Vaccine L, Middle dose of candidate vaccine=Vaccine M. Asterisks denote significant differences between the adjuvant (placebo) and control (PBS) group and the vaccine groups (Vaccine L, Vaccine M and Vaccine H).


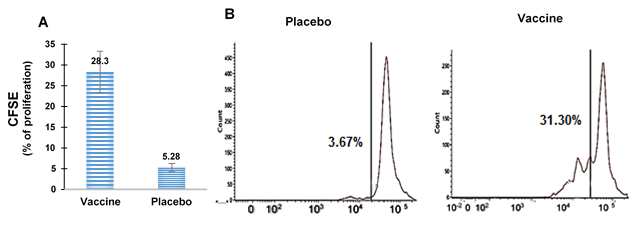


Figure 7S: CFSE proliferation assay of MNCs derived from mice spleens. A) MNCs in vaccinated group showed higher proliferative capacity in response to S specific antigens compared to placebo control group (28.30±5% of cells relative to 5.28±1.70%). Data are mean±SD analyzed with non-parametric Mann Whitney test, p<0.05. B) Representative flow cytometry plots from animal immunized with middle dose of vaccine and placebo control illustrated.


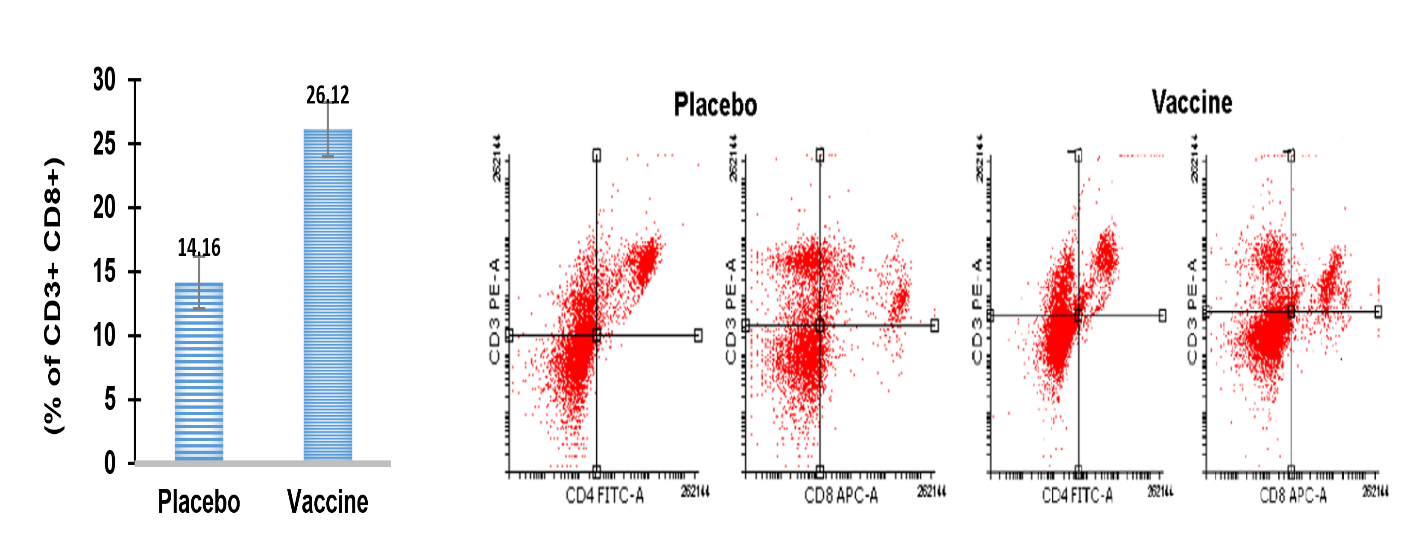


Figure 8S. **Flow cytometric analysis of CD3/CD4/CD8 markers in MNCs derived from mice spleens.** As illustrated in panel A, CD3+ CD8+ cells significantly increased in MNCs in vaccinated group in response to S specific antigens compared to placebo control group (26.12% relative to 14.16 ±%). Data are mean±SD analyzed with non-parametric Mann Whitney test, p<0.05. B) Representative plots from animal immunized with middle dose of vaccine and placebo control (adjuvant) group illustrated. (p<0.05).


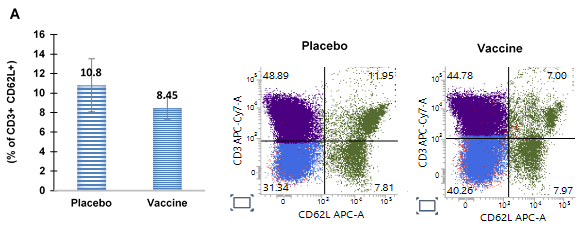


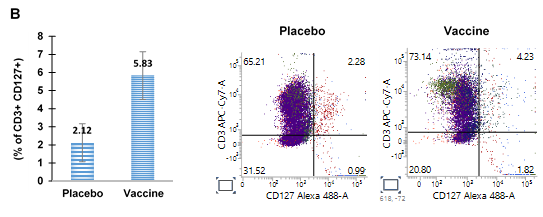


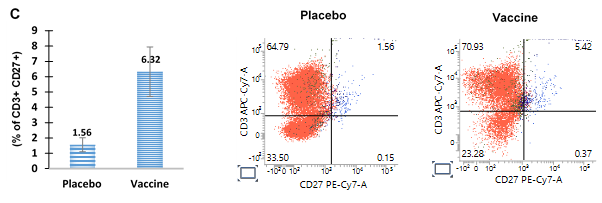


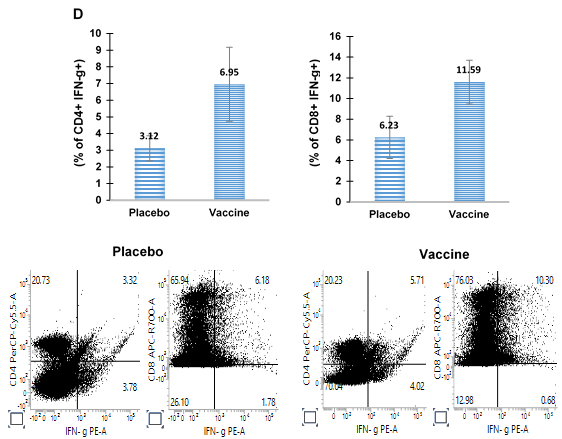


Figure 9S. Flow cytometric analysis of activation/memory surface markers. CD3+ MNCs derived from mice spleen of vaccinated group rather than placebo group (adjuvant) showed; A) reduced expression of CD62L, B) higher expression of CD127 derived from mice spleen of vaccinated group rather than placebo group. C) higher expression of CD27 derived from mice spleen of vaccinated group rather than placebo group D) CD4+ and CD8+ MNCs expressed more intra cellular IFNγ cytokine. Data are mean ±SD analyzed with non-parametric Mann Whitney test, p<0.05. B). Moreover, representative plots from animal immunized with middle dose of vaccine and placebo control (adjuvant) group were illustrated (p<0.05).
